# Dietary organosulfur compounds: Emerging players in the regulation of bone homeostasis by plant-derived molecules

**DOI:** 10.3389/fendo.2022.937956

**Published:** 2022-09-15

**Authors:** Laura Gambari, Brunella Grigolo, Francesco Grassi

**Affiliations:** Laboratorio RAMSES, IRCCS Istituto Ortopedico Rizzoli, Bologna, Italy

**Keywords:** organosulfur compounds (OSCs), osteoporosis, hydrogen sulfide (H_2_S), *Brassicaceae*, *Allium*, glucosinolates, isothiocyanates, polysulfides

## Abstract

The progressive decline of bone mass and the deterioration of bone microarchitecture are hallmarks of the bone aging. The resulting increase in bone fragility is the leading cause of bone fractures, a major cause of disability. As the frontline pharmacological treatments for osteoporosis suffer from low patients’ adherence and occasional side effects, the importance of diet regimens for the prevention of excessive bone fragility has been increasingly recognized. Indeed, certain diet components have been already associated to a reduced fracture risk. Organosulfur compounds are a broad class of molecules containing sulfur. Among them, several molecules of potential therapeutic interest are found in edible plants belonging to the *Allium* and *Brassica* botanical genera. Polysulfides derived from *Alliaceae* and isothiocyanates derived from *Brassicaceae* hold remarkable nutraceutical potential as anti-inflammatory, antioxidants, vasorelaxant and hypolipemic. Some of these effects are linked to the ability to release the gasotrasmitter hydrogen sulfide (H_2_S). Recent preclinical studies have investigated the effect of organosulfur compounds in bone wasting and metabolic bone diseases, revealing a strong potential to preserve skeletal health by exerting cytoprotection and stimulating the bone forming activity by osteoblasts and attenuating bone resorption by osteoclasts. This review is intended for revising evidence from preclinical and epidemiological studies on the skeletal effects of organosulfur molecules of dietary origin, with emphasis on the direct regulation of bone cells by plant-derived polysulfides, glucosinolates and isothiocyanates. Moreover, we highlight the potential molecular mechanisms underlying the biological role of these compounds and revise the importance of the so-called ‘H_2_S-system’ on the regulation of bone homeostasis.

## Highlights

A literature search was conducted using MEDLINE database. Relevant pre-clinical and clinical studies were selected using a combination of keywords including bone, diet and/or organosulfur compounds, *Allium*, *Brassicaceae*, alliin, allicin, garlic, ajoene, diallyl trisulfide, diallyl disulfide, *S*-allylcysteine, diallyl sulfide, glucosinolate, thiosulfinate, sulforaphane, broccoli, methyl sulfide, isothiocyanates. Additional studies were identified by an extensive manual search of bibliographic references in original papers and reviews. Abstracts and non-English papers were not included. This study selected a total of *in vitro* studies (10 *Alliaceae*, 9 *Brassicaceae*); *in vivo* studies (17 *Alliaceae*, 11 *Brassicaceae*) and population-based studies (4 *Alliaceae*, 1 *Brassicaceae*).

## Introduction

Osteoporosis (OP) is a chronic metabolic bone disease characterized by the deterioration of bone microarchitecture and a reduction in bone mass, leading to decreased bone strength and increased risk of bone fracture (1). Approximately 6 % of men and 21 % of women aged 50–84 years are diagnosed with OP and the number of fragility fractures in Europe has increased from 3.1 to nearly 4.3 million in 20 years since year 2000 (2); due to the strong correlation with the ageing of the population, the prevalence of OP is projected to further increase over the next decades (3).

At the bone tissue level, OP is characterized by increased bone porosity which results from the loss of balance between bone formation and bone resorption as aging, disuse, inflammatory diseases, hormonal imbalance or the effect of glucocorticoids impair the ability of osteoblast to keep up with the pace of bone resorption by the osteoclasts (4). Importantly, aging is associated with a decreased number of osteoprogenitor cells, inhibited proliferation, decreased mineralizing capacity, and a shift of osteogenic differentiation toward adipogenesis in senescent mesenchymal stromal cells (MSCs) (5–7).

Pharmacotherapy helps patients to prevent the occurrence or recurrence of fragility fractures and to manage symptoms. However, drugs are mostly used in patients who already show severe bone loss, and the existence of side effects, although very limited in prevalence, often leads to low patient’s adherence to anti-OP drugs (8, 9). In this context, non-pharmacological strategies aimed at preventing excessive bone loss hold relevance given that OP remains in most cases a subclinical condition until fracture occurs.

One safe way to prevent bone loss and reduce the risk of bone fracture is to positively impact bone mass through healthy lifestyles and nutrition (10, 11). In particular, the importance of defining specific diet regimens for the prevention of excessive bone fragility has been increasingly recognized (12–15). Adherence to Mediterranean diet lowered hip fracture risk (16) and certain micronutrients contained in fruit and vegetables contributed to delay bone fragility in ageing and to decrease the incidence of bone fractures (17–20). Moreover, a dietary pattern consisting of a high consumption of fruits, vegetables and seafood, has been shown to be directly associated with increased bone mineral density (BMD), independent of dietary calcium intake (21, 22).

Phytochemicals are defined as the chemical bioactive components of nutrient plants that may provide desirable health benefits beyond basic nutrition to reduce the risk of major chronic diseases. They include several classes of compounds: terpenoids, polyphenols, alkaloids, organosulfur compounds (OSCs) and phytosterols (23). Concerning OSCs, much of the research on their health benefits has been in the areas of cardiovascular diseases, cancer and neurological disorders (24–26). However, a growing body of scientific evidence supports the idea that dietary OSCs may play an important role for skeletal health by favoring bone anabolism, inhibiting bone catabolism, and preventing pathological bone loss.

This manuscript intends to provide an up-to-date review of the current evidence from preclinical (both *in vitro* and *in vivo*) and clinical studies on the skeletal effects of OSCs of dietary origin, discussing the chemical nature, the mechanism of action and the potential role of hydrogen sulfide (H_2_S) in their biological action. A specific focus is given to the pair glucoraphanin (GRA)-sulforaphane (SFN) as a paradigm of OSCs-H_2_S system in bone tissue. Finally, implications and future challenges in the field will be discussed considering the potential translation of OSCs-containing dietary components to clinical studies.

## Dietary sources and chemical nature of OSCs

Naturally derived OSCs are a broad class of molecules containing sulfur, predominantly found in edible plants belonging to the *Allium* and *Brassica* (also known as cruciferous vegetables) genera. These plants have been widely used throughout the centuries either as vegetables for culinary purposes as well as in folk and traditional medicine, given their renowned medicinal properties and therapeutic effects. *Allium* genus consists of more than 600 species which are among the oldest cultivated vegetables used as food and still represent one of the main components of the Mediterranean diet (27). *Brassica* genus consists of 37 species; among them, several species are known for their nutritional and therapeutic properties (28, 29). A partial list of edible plants belonging to the *Allium* and *Brassica* genera, and their main content in OSCs, is reported in **Table 1**.

**Table 1:** Most common OSCs found in edible *Allium* and *Brassica* vegetables.

Edible plants	Genus	Main OSCs			REF
**Garlic (*Allium sativum L.*)**	*Allium*	• γ-glutamyl-*S*-allyl-l-cysteine • allicin • alliin • methiin *• S*-*trans*-1-propenylcysteine sulfoxide *• S*-2-carboxypro-pylglutathione *• S*-allylcysteine • ajoene • vinyldithiins • diallyl sulfide • diallyl disulfide • diallyl trisulfide		*• S*-allylcysteine *• S*-allylmercaptocysteine *• S*-allylmercaptoglutathione • methyl allyl disulfide • methyl allyl trisulfide *• S*-allylmercaptocysteine • dipropyl disulfide • dipropyl trisulfide • 1-propenylpropyl disulfide • dimethyl disulfide • allyl mercaptan • propyl propane thiosulfonate	(30–39)
**Onion (*Allium cepa L*.)**	*Allium*	• isoalliin • methiin • propiin • diallyl disulfide • diallyl trisulfide • γ-l-glutamyl-trans-*S*-1-propenyl-l-cysteine sulfoxide		• onionin A • cycloalliin *• S*-methyl cysteine sulfoxide *• S*-propenyl cysteine sulfoxide *• S*-alk(en)yl cysteine sulfoxides • dipropyl disulfide • cycloalliin	(40–45)
**Welsh onion (*Allium fistulosum* L.)**	*Allium*	• γ-glutamyl-*S*-allyl-l-cysteine • allicin		• alliin • diallyl disulfide	(46, 47)
**Hooker’s Onion (*Allium hookeri*)**	*Allium*	• alliin • methiin		• cycloalliin *• S*-propyl-l-cysteine sulfoxide	(48–50)
**Long-stamen chive (*Allium macrostemon*)**	*Allium*	• alliin • methyl alliin			(51)
**Leek (*Allium ampeloprasum* var. *porrum*)**	*Allium*	• methiin • isoalliin			(52)
**Shallot (*Allium ascalonicum*)**	*Allium*	• isoalliin • methiin		• propiin • γ-glutamyl-*S*-alk(en)ylcysteines	(53)
**Turnip (*Brassica rapa L.*)**	*Brassica*	• glucoraphanin & sulforaphane • gluconapin & 3-butenyl isothiocyanate • glucobrassicanapin & 4-pentenyl isothiocyanate/gluconapoleiferin • gluconasturtiin & 2-phenethyl isothiocyanate • goitrin • berteroin		• progoitrin • glucoalyssin • glucoerucin • glucobrassicin & 4-hydroxyglucobrassicin/4-methoxyglucobrassicin • glucoberteroin • neoglucobrassicin	(54, 55)
**Broccoli (*Brassica oleracea var. italica L.*)**	*Brassica*	• sulforaphane • glucoiberin • 3-hydroxy,4(α-L-rhamnopyranosyloxy) benzyl glucosinolate			(56–58)
**Water cress (*Lepidum sativum L.*)**	*Brassica*	• glucotropaeolin			(59)
**Cabbages (*Brassica oleracea var. capitata L.*)**	*Brassica*	• glucoraphanin • progoitrin • sinigrin • gluconapin • glucoerucin	• glucobrassicin & 4-hydroxyglucobrassicin/• 4-methoxyglucobrassicin • neoglucobrassicin • glucoiberin		(60, 61)
**Rocket (*Eruca sativa*)**	*Brassica*	• glucoraphanin • glucoraphenin • glucosativin • glucoerucin • 4-hydroxyglucobrassicin • glucotropaeolin	• glucolepiidin • glucoiberverin • glucoalyssin • diglucothiobeinin • glucoibarin		(62)
**Kohlrabi *(Brassica oleracea* var. *gongylodes)***	*Brassica*	• glucoraphanin & sulforaphane • glucoerucin & methylthiobutyl isothiocyanate • benzyl isothiocyanate • gluconasturtiin & phenylethyl isothiocyanate	• sinigrin & allyl isothiocyanate • glucobrassicin & hydroxyglucobrassicin • neoglucobrassicin • methiin		(63, 64)
**Radish *(Raphanus sativus)***	*Brassica*	• 3-butenyl isothiocyanate • glucobrassicin/4-methoxyglucobrassicin/4- hydroxyglucobrassicin/indole-3-carbinol	• glucodehydroerucin • glucoraphasatin • glucoraphenin/sulforaphene • sulforaphane		(65)
**Tuscan black kale *(Brassica oleracea L.*)**	*Brassica*	• glucoerucin • glucobrassicin • glucoraphanin			(66)
**Rapes (*Brassica napus L.*)**	*Brassica*	• glucoalyssin • glucobrassicin & hydroxyglucobrassicin • neoglucobrassicin	• gluconasturtin • gluconapin • glucobrassicanapin • progoitrin		(61, 67)
**Arugula (*Eruca Sativa Mill.*)**	*Brassica*	• glucoraphanin & sulforaphane	• glucoerucin & erucin		(68)

In *Allium*, over half of the total sulfur content within the mature garlic bulb is found in the form of *S*-alk(en)yl cysteine sulfoxides (ASCOs) (69), non-protein sulfur amino acids which are converted to their respective thiosulfinates or propanethial-*S*-oxide upon tissue damage (70).

The synthesis of ASCOs in *Allium* species starts with the transformation of γ-glutamyl peptides (such as γ-l-glutamyl-*S*-methyl-L-cysteine) into sulfur-containing γ-glutamyl-*S*-alk(en)yl-cysteines such as γ-glutamyl-*S*-methyl-cysteines, γ-glutamyl-*S*-allyl-cysteine, γ-glutamyl-propenyl-l-cysteine sulfoxide (PeCSO). These are further deglutamylated and *S*-oxygenated to yield *S*-alk(en)yl-l-cysteine sulfoxides (71, 72). These reactions are catalyzed by γ-glutamyl transpeptidase, l-glutaminases, and oxidase in the cytoplasm of plant cells. The intact garlic bulbs contain alliin, γ-glutamyl-*S*-allyl-l-cysteine (GSAC), methiin, *S*-*trans*-1-propenyl-l-cysteine sulfoxide, *S*-2-carboxypropylglutathione, *S*-allylcysteine (SAC) (37).

When the bulbs are cut, crushed, chopped or chewed, the enzyme alliinase (a vacuolar lyase) is released from vacuoles and catalyzes the formation of sulfenic acids from l-cysteine sulfoxides: *S*-allyl-l-cysteine sulfoxide (alliin); *S*-methyl-l-cysteine sulfoxide (methiin); *S*-propyl-l-cysteine sulfoxide (propiin); *S*-*trans*-1-propenyl-l-cysteine sulfoxide (isoallin) (71, 72). Sulfenic acids spontaneously react with each other to form unstable compounds called thiosulfinates (69): eg. alliin is converted into allicin (alkenyl alkene thiosulfinate - diallyl thiosulfinate). Allicin immediately decomposes into allyl sulfide (AS), diallyl disulfide (DADS), diallyl trisulfide (DATS), diallyl tetrasulfide, dipropyl disulfide (DPDS), ajoenes, and vinyldithiins (72). The direct catabolism of γ-glutamylcysteine by γ-glutamyltranspeptidase leads to the formation of SAC and *S*-allylmercaptocysteine (SAMC). Allicin can react with glutathione and l-cysteine to produce *S*-allylmercaptoglutathione (SAMG) and SAMC, respectively (69, 72).

Among *Allium*, the most common ASCOs are alliin, methiin, propiin and isoalliin (70, 73, 74). However, they are differentially expressed in specific edible plants. The most abundant in garlic is alliin; in onion isoalliin, methiin, propiin are predominantly detected.

In *Brassica* vegetables two different kinds of OSCs are present: methiin, mainly known from *Allium* vegetables, and glucosinolates (S-β-thioglucoside N-hydroxhysulfates, GLS). Methiin is metabolized to (+)-*S*-alk(en)yl-l-cysteine sulfoxides which can degrade to volatile organosulfur compounds (VOSCs) such as *S*-methyl methane thiosulfinate, which is converted to dimethyl trisulfide and dimethyl disulfide.

GLS are sulfur-based compounds that consist of β-thioglycoside N-hydroxysulfates with various side chains and a sulfur-linked β-d-glycopyranose moiety. A very different profile of GLS may be found in different *Brassica* extracts (75). Natural isothiocyanates (ITCs) are bioactive OSCs derived from the hydrolysis of GLS by the enzyme myrosinase. In plant cells, GLS are physically separated from myrosinases and come in contact only upon tissue damage or crushing. Importantly, myrosinase is not expressed by mammalian cells; however, a small proportion is converted in the mouth by action of plant myrosinase released by chewing (76); moreover, the gut microbiota is entailed with myrosinase activity and constitutes the major site in humans where GLS are hydrolyzes to ITCs (77). While GLS are chemically stable and are characterized by a relatively long half-life, ITCs are highly reactive and short-lived *in vivo* (75, 78).

## Effect of OSCs on bone tissue: Preclinical evidence

The effect of OSCs in bone tissue has been investigated in several preclinical models, revealing a strong potential to preserve skeletal health by stimulating the bone forming activity of osteoblasts and inhibiting the bone resorbing activity of osteoclasts, two of the key processes of bone remodeling (79).

**Figure 1:**
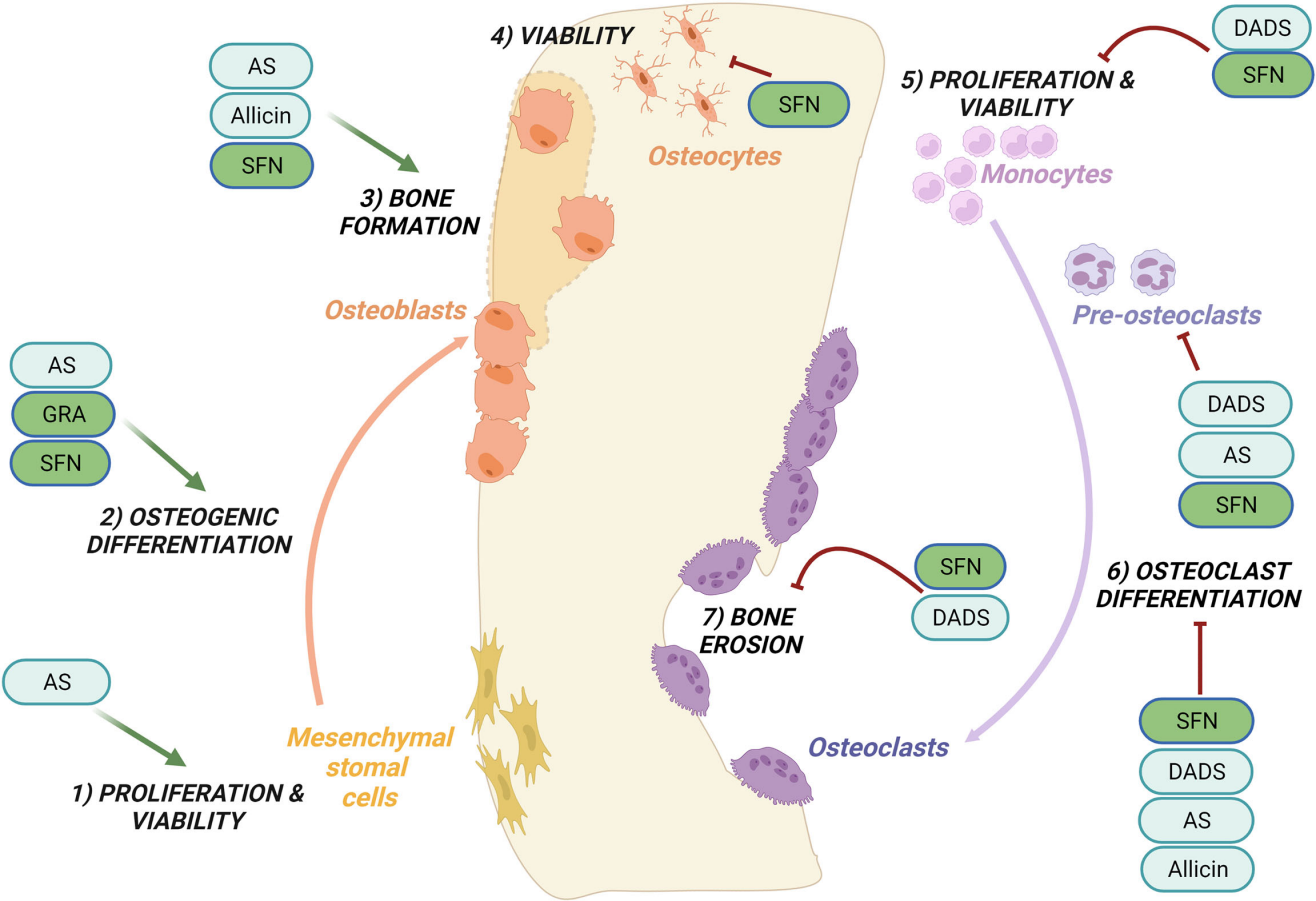
Regulation of bone remodeling processes by purified OSCs molecules. Bone remodeling is governed by the balance between bone formation by the osteoblasts (left side) and bone erosion by the osteoclasts (right side). Ancillary processes are shown. OSCs specifically regulate the following processes: promote cells proliferation and viability of mesenchymal stromal cells (1) while inhibit the proliferation and viability of monocytes (5); promote the osteogenic differentiation (2) and bone formation (3); inhibit at different stages osteoclast differentiation (6) and reduce bone erosion (7); inhibit the viability of osteocytes (4). Among the OSCs which modulate bone processes are allicin, allyl sulfide (AS), sulforaphane (SFN), glucoraphanin (GRA), diallyl sulfide (DADS). See the text for details.

**Figure 2:**
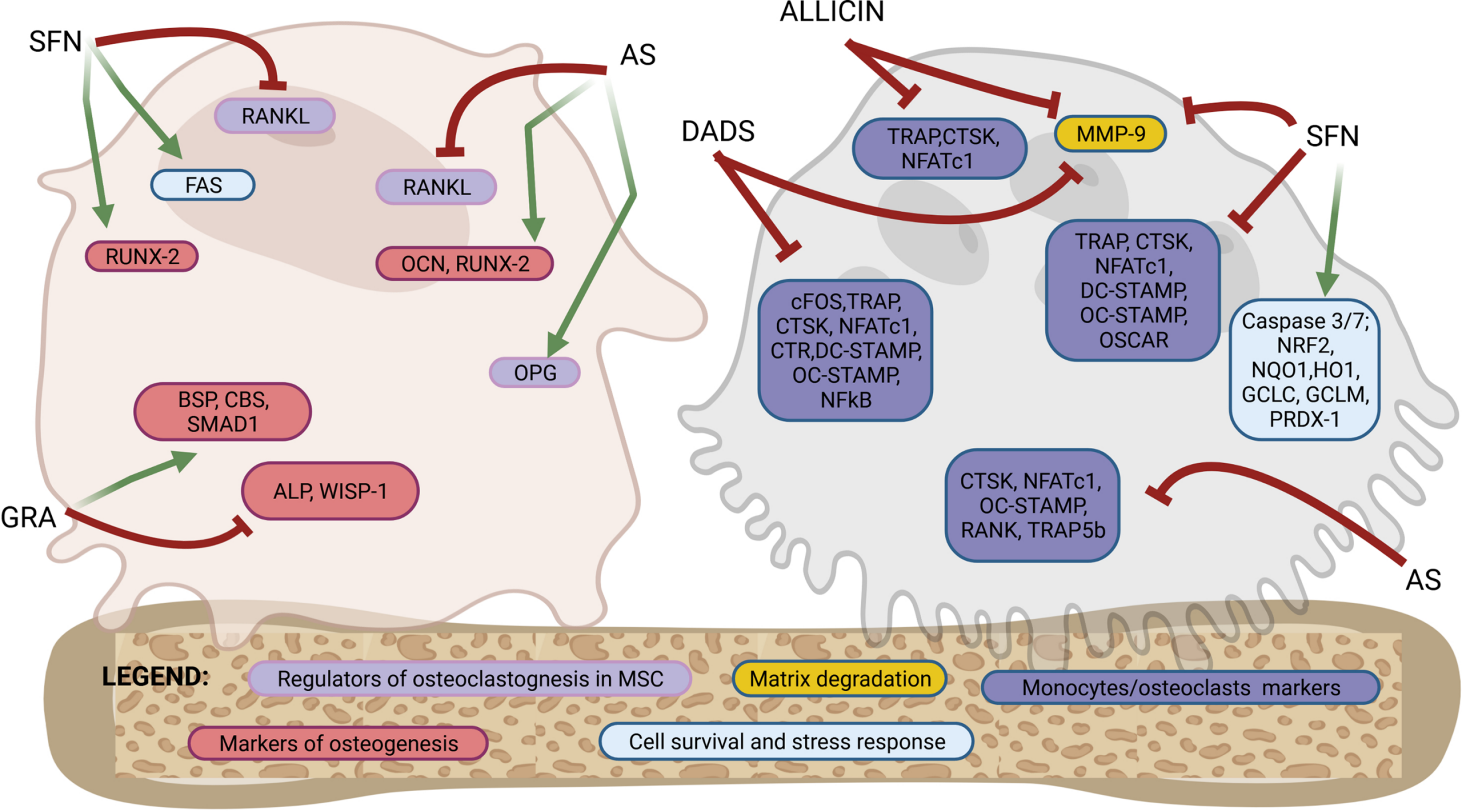
Molecular targets of purified OSCs molecules in bone cells. Osteoblastogenesis and osteoclastogenesis are the two key processes of bone remodeling and are regulated by a tightly organized activation of specific molecular targets. This figure shows a schematic representation of a mesenchymal stromal cells/osteoblast and a monocyte/osteoclast to highlight the specific molecular targets regulated by OSCs at different stages of differentiation from precursors to fully differentiated cells. Among the OSCs which drives the modulation of specific molecular targets are allicin, allyl sulfide (AS), sulforaphane (SFN), glucoraphanin (GRA) and diallyl sulfide (DADS). The overall effects are an activation of osteogenic differentiation in mesenchymal stromal cells and both a direct and indirect inhibition of osteoclast differentiation. Follows a list of the molecular targets shown in the figure. Markers of osteoblastogenesis: osteocalcin (OCN), runt-related transcription factor 2 (RUNX-2), alkaline phosphatase (ALP), WNT1-inducible-signaling pathway protein 1 (WISP-1), bone sialoprotein (BSP), cystathionine-β-synthase (CBS), SMAD family member 1 (SMAD-1). Markers of regulators of osteoclastogenesis produced by mesenchymal stromal cells or osteoblasts: receptor activator of nuclear factor-κB ligand (RANKL), osteoprotegerin (OPG). Marker of cells survival and stress response: FAS, caspase 3/7, nuclear factor erythroid-derived 2-related factor 2 (NRF2), NAD(P)H: quinone oxidoreductase 1 (NQO1), heme oxygenase-1 (HO1), glutamate cysteine ligase catalytic subunit (GCLC), glutamate-cysteine ligase modifier subunit (GCLM), peroxiredoxin 1 (PRDX-1). Markers of osteoclasts: nuclear factor of activated T-cells cytoplasmic 1 (NFATc1), cathepsin K (CTSK), receptor activator of NF-KB (RANK), osteoclast stimulatory transmembrane protein (OC-STAMP), dendritic cell specific transmembrane protein (DC-STAMP), osteoclasts-specific activating receptor (OSCAR), tartrate-resistant acid phosphatase (TRAP), calcitonin receptor (CTR), c-fos, tartrate-resistant acid phosphatase 5b (TRAP-5b), matrix metallopeptidase 9 (MMP-9). See the text for details.

**Figures 1**, **2** provide a graphical summary, respectively, of the main biological processes and molecular targets regulated by OSCs within MSCs/osteoblasts and monocytes/osteoclasts. A detailed description of these mechanisms is provided in the next paragraphs.

**Tables 2**–**5** summarize data from preclinical studies showing an effect of extracts rich in OSCs or individual OSCs molecules derived from *Allium* (**Tables 2**, **3**) and *Brassica* vegetables (**Tables 4**, **5**).

**Table 2:** *Alliaceae-*derived OSCs: effects on *in vitro* models of osteoclastogenesis and osteoblastogenesis.

Molecule tested	Experimental *in vitro* model	Concentration	Main effect	Specific outcomes	Authors	Ref
**Hot-water extract and ethanol extracts of *Allium hookeri* roots**	MG-63 cells line	0.1-0.5-1-5-10-25-50-100 μg/ml	Increased proliferation and osteogenesis	• ↑ viability/proliferation; no cytotoxicity (WST-8 assay) • ↑ ALP activity (pNPP detection) • ↑ collagen (Sirius red assay) • ↑ mineralization (Alizarin Red staining)	Park et al.	(48)
**Aqueous and ethanolic extracts of *Allium fistulosum***	MG-63 cell line	1-4-8-10-16-32-50-63-125 μg/ml	Increased osteogenesis	• no cytotoxicity (MTT assay) • ↑ ALP activity (ALP assay kit)	Ryuk et al.	(80)
**Water solution of onion crude powder**	MG-63 cell line	300 μg/ml	No effect on proliferation or differentiation	• ALP activity similar to control cells (ALP assay kit) • Col I on cell lysate was similar to control cells (4- hydroxyproline quantification) • OCN, OPN in cells surnatants similar to control cells (ELISA)	Tang et al.	(81)
**Aqueous and ethanolic extracts of *Allium fistulosum***	MC3T3-E1 cell line	1-4-8-10-16-32-50-63-125 μg/ml	Increased proliferation and osteogenesis	Ethanolic extracts: • ↑ viability/proliferation; no cytotoxicity (MTT assay) • ↑ALP activity (ALP assay kit) Water extracts: • no cytotoxicity (MTT assay) • ↑ALP activity (ALP assay kit)	Ryuk et al.	(80)
**Water *Allium sativum L.* extract**	Human fetal osteoblast cells	3D-printed calcium phosphate scaffolds releasing ginger and garlic extract	Increased osteoblast proliferation	• ↑ proliferation (MTT assay)	Bose et al.	(82)
Allyl sulfide (AS) *	BMMSCs isolated from Age-associated OP mice’s femurs	Mice were fed by oral gavage with AS (200 mg/kg) for 3-months	• Rescue of proliferation and osteogenesis • Indirect inhibition of osteoclastogenesis	• ↑ proliferation as compared to aged mice (MTT assay) • ↑ALP activity (ALP staining), ↑ mineralization (Alizarin red staining), • ↑ RUNX-2 and OCN in cells (western blot) • ↑ OPG and ↓ RANKL in surnatants (ELISA)	Behera et al.	(83)
***Allium cepa* L. extracts**	*In vitro* bioactivity assay (simulated body fluid)	Chitosan + *Allium cepa L.* (ChAC) and Chitosan + *Allium cepa L.* + PLGA (ChPAC)	Improved natural bioactivity of chitosan	• Increased apatite cristals in the surface • Improved Phosphorous/Calcium ratio	Monárrez-Cordero et al.	(84)
**Water *Allium sativum L.* extract**	Human osteoclast cells from THP1 monocytes	3D-printed calcium phosphate scaffolds releasing ginger and garlic extract	Inhibition of osteoclast activity	• ↓ resorption (pit assay)	Bose et al.	(82)
**Ethanolic extract of** **onion**	RAW 264.7 cell line	0.1-0.2-0.4 mg/ml	Inhibition of osteoclastogenesis	• no cytotoxicity (MTT assay) • ↓ osteoclasts (TRAP assay)	Law et al.	(85)
**Freeze dried onion juice**	RAW 264.7 cell line	0.1-0.2-0.4 mg/ml	Inhibition of osteoclastogenesis	• no cytotoxicity (MTT assay) • ↓ osteoclasts (TRAP assay)	Law et al.	(85)
**Water solution of onion crude powder**	RAW 264.7 cell line	15-50-150-300 μg/ml	Inhibition of osteoclastogenesis	• no cytotoxicity (MTT assay) • ↓ osteoclasts (TRAP assay) • ↓ CD51/61 (vitronectin receptor), MMP-9 and TRAP mRNA (RT-PCR) • ↓ ERK, p38 and NF-κB (western blot)	Tang et al.	(81)
Diallyl disulfide (DADS) *	RAW 264.7 cell line	1-10-100-1000 μg/ml 20-40-60-80-100 μg/ml	Inhibition of osteoclastogenesis and bone resorption	• ↓ cytotoxicity at concentration higher to 100 μg/ml (CCK-8 assay) • ↓ osteoclast and resorption (TRAP assay PIT assay) • ↓ c-fos, NFATc1, TRAP, MMP9, CTR, CTSK, DC-STAMP, OC-STAMP mRNA • ↓ osteoclast fusion (FAK staining) • ↓ NF-ĸB, p-STAT3, NFATc1, c-FOS (western blot)	Yang et al.	(86)
Alliin *	RAW 264.7 cell line	0.1-0.5-1-5-10-100 μg/ml	Inhibition of osteoclastogenesis	• No cytotoxicity (CCK-8 assay) • ↓ osteoclasts and resorption (TRAP assay and pit assay) • ↓ c-fos, NFATc1, MMP9, DC-STAMP, OC-STAMP, RANK, TRAP (RT-PCR) • ↓ Nox-1, NFATc1, c-fos (western blot) • ↓ ROS (detection by fluorescent probe)	Chen et al.	(87)
**Water solution of onion crude powder**	Osteoclast derived from bone marrow cells of femurs of 6-8-week-old Sprague–Dawley rats	15-50-150-300 μg/ml	Inhibition of osteoclastogenesis	• no cytotoxicity (MTT assay) • ↓ osteoclasts (TRAP assay)	Tang et al.	(81)
**Water solution of onion crude powder**	Osteoclast derived from long bones of 6-day-old rabbits	15-50-150-300 μg/ml	Inhibition of bone resorption	• ↓ resorption (pit assay)	Tang et al.	(81)
**Commercial onion powder (Chia Hui, Taipei, Taiwan)**	Osteoclast derived from bone marrow cells of femurs of 6-8-week-old Sprague–Dawley rats	300 μg/ml	Inhibition of osteoclastogenesis	• ↓ osteoclasts (TRAP assay) • Inhibition of ERK, p38, and NF-κB activation (western blot)	Tang et al.	(81)
**GPCS isolated by bioassay-guided fractionation of** *Allium cepa L.* Bulbs *	Osteoclasts derived from femora and tibiae of 2-days-old Wistar Hanlbm rats	1-10-30 mg/ml 2-4-8 mM	Inhibition of osteoclast differentiation and activity	• ↓ osteoclast differentiation and resorption by GPCS (TRAP staining and pit assays)	Wetli et al.	(41)
Diallyl disulfide (DADS) *	BMMs obtained from the femur and tibia bone marrow of 6-wk-old C57BL/6 mice	20-40-60-80-100 μg/ml	Inhibition of osteoclastogenesis	• ↓ cytotoxicity at concentration higher to 100 μg/ml (CCK-8 assay) • ↓ osteoclast (TRAP assay)	Yang et al.	(86)
Allyl sulfide (AS)*	BM cells	Cultured under 15% conditioned medium derived from BMMSCs culture of Age-associated OP mouse model (Fed by oral gavage with AS (200 mg/kg) for 3-months)	Inhibition of osteoclastogenesis *via* a paracrine mechanism	• ↓ osteoclasts (TRAP staining) • ↓ TRAP-5b expression in cells lysates (ELISA) • ↓ NFATc1, CTSK, RANK and OC-STAMP mRNA (RT-PCR)	Behera et al.	(83)

Most *in vitro* studies were conducted by using water or ethanol extracts from *Allium* edible plants (4 studies, 13 *in vitro* models; *Allium* hookeri roots, *Allium fistulosum*, *Allium sativum L., Allium cepa L.*); a few used purified OSCs (3 studies, 6 *in vitro* models; diallyl disulfide (DADS), allyl sulfide (AS), *γ*-glutamyl-*trans*-S-1-propenyl-L-cysteine sulfoxide – GPCS, alliin). Most studies showed an increased osteoblast proliferation and osteogenesis and an inhibited osteoclastogenesis. Notably, only the effects of purified OSCs (labeled with * in the table) can be attributable entirely to OSCs. The concentrations tested ranged from 0.1 to 300 μg/ml. Murine *in vitro* models of osteoclastogenesis: osteoclasts derived from bone marrow of femora and tibiae of rats, rabbits, mice; RAW 264.7 cells. Human *in vitro* models of osteoclastogenesis: osteoclast cells from human THP1 monocytes. Murine *in vitro* models of osteoblastogenesis used: MC3T3-E1 (mouse C57BL/6 calvaria cells line); murine bone marrow (BM) cells; bone marrow-derived mesenchymal stem cells (BMMSCs) isolated from age-associated (AG) osteoporosis (OP) mice’s femurs. Murine in vitro models for studying indirect inhibition of osteoclastogenesis: bone marrow-derived mesenchymal stem cells (BMMSCs), bone marrow macrophages (BMM) and murine bone marrow (BM). Human *in vitro* models of osteoblastogenesis: MG-63 cells line (human osteosarcoma cells line), human fetal osteoblast. Functional assays for osteoclastogenesis used: tartrate-resistant acid phosphatase positive (TRAP staining); pit assay. Functional assays for osteoblastogenesis: alizarin red staining (marker of mineralization), sirius red assay (marker of collagen I), *p*-nitrophenyl phosphate (pNPP) measurement. Proliferation/viability assays: 3-(4,5-Dimethylthiazol-2-yl)-2,5-diphenyltetrazolium bromide (MTT) assay, cell counting kit-8 (CCK-8) cell viability assay, water-soluble tetrazolium-8 (WST-8) assay. Markers of osteoclasts: nuclear factor of activated T-cells cytoplasmic 1 (NFATc1), cathepsin K (CTSK), receptor activator of NF-KB (RANK), osteoclast stimulatory transmembrane protein (OC-STAMP), tartrate-resistant acid phosphatase (TRAP), tartrate-resistant acid phosphatase 5b (TRAP-5b), receptor activator of nuclear factor-κB ligand (RANKL), dendritic cell specific transmembrane protein (DC-STAMP), reactive oxygen species (ROS), calcitonin receptor (CTR), p-signal transducer and activator of transcription 3 (p-STAT3), NADPH Oxidase 1 (Nox-1), c-fos, nuclear factor kappa-light-chain-enhancer of activated B cells (NF-κB), p38, extracellular signal-regulated kinase (ERK), matrix metallopeptidase 9 (MMP-9), CD51/61 (vitronectin receptor). Markers of osteoblastogenesis: collagen I (Col I), osteocalcin (OCN), osteopontin (OPN), runt-related transcription factor 2 (RUNX-2), osteoprotegerin (OPG), alkaline phosphatase (ALP). ↑ means up-regulation; ↓ means down-regulation.

**Table 3:** *Alliaceae-*derived OSCs: effects on *in vivo* models of bone loss.

Molecule tested	Experimental *in vivo* model description	Mode of administration, dose and duration	Main effect	Specific outcomes	Authors	Ref
**Ethanol extracts of *Allium macrostemon* bulbs**	Female, 25-day-old, Sprague–Dawley rats (adolescent mice)	Gavage, 100 and 300 mg/kg, twice daily for 10 days	Increase tibial longitudinal bone growth	Increase tibial longitudinal bone growth (fluorescence photomicrograph after tetracycline hydrochloride) ↑ IGF-1 and BMP-2 in the proliferative and hypertrophic zones of growth plate (immunohistochemistry)	Kim et al.	(85)
**Hot-water extracts of *Allium hookeri* roots**	Female, 3-week-old, Sprague-Dawley rats	Oral treatment, 500 mg/kg, single daily dose, for 6 weeks	Improved bone formation	↑ serum levels of OCN (ELISA) ↑ BMD, BV, BV/TV, Tb.Th, Tb.N; ↓ Tb.Sp, BS/BV (microCT in proximal tibia)	Park et al.	(48)
**Wheat bread added with *Allium sativum L.***	Male weaning Wistar rats	Oral administration, 3 g per 100 g wheat flour, for 60 days	Increase in BMD	↑ total skeleton BMC and BMD, femur BMD, tibia BMD Spine (S-BMD) and proximal tibia (T-BMD) was not affected (DEXA) ↑ femur calcium	Weisstaub et al.	(88)
**Ethanolic extracts of *Allium cepa L.* bulbs**	Male, 9-week-old, Wistar Hanlbm rats	Orally given, one gram, daily treatment, for 10 days	Inhibition of bone resorption	↓ bone resorption (urinary excretion of tritium)	Wetli et al.	(41)
**Homogenized of *Allium sativum L.***	Hypercholesterolemic rat model (Pregnant albinorat Wistar fed with hypercholesterolemic diet, and their offspring)	Intragastrical injection, 100 mg/kg, a week prior to onset of feeding with hypercholesterolemic diet	Improved endochondral ossification	↑ ossification in mandibular, humerus, radio-ulna, femur, tibio-fibula, scapula and ilium (Alizarin red S for ossified skeletal bones in fixed offspring)	El-Sayyad et al.	(89)
**Water *Allium sativum L.* extract**	*In vivo* implants in bicortical rat distal femur defects (Sprague–Dawley rats)	3D-printed calcium phosphate scaffolds designed with a bimodal pore distribution releasing ginger and garlic extract, implanted for 4-10 weeks	Increase in osteoinductivity	↑ osteoid tissue formation, mineralization (masson-goldner trichrome assay) ↑ bone area, osteocytes (haematoxylin and eosin) ↑ Col I (Col I staining)	Bose et al.	(82)
**Aqueous and ethanolic extracts of *Allium fistulosum***	CDD mice - Mice model of bone loss due to nutritional deficiency (Male, 4-week-old, C57BL/6 mice, fed with a calcium- and vitamin D-deficient diet for 5 weeks)	Oral treatment, 150 and 450 mg/kg, ad libitum feeding for 4 weeks	Prevention nutritional deficiency-induced bone loss and retarded bone growth	↑ serum calcium, OC and Col I *vs* CDD mice (ELISA) ↑ serum ALP, OCN and Col I *vs* normal control mice (ELISA) ↑ femoral and tibial BMC and BMD *vs* CDD mice and similar to normal control (DEXA) Thicker growth plates *vs* CDD mice and similar to normal control (measured after hematoxylin and eosin stain)	Ryuk et al.	(80)
**Water extract of *Allium fistulosum* root**	Rat model of OP and osteoarthritis (Female, 8-week-old, Sprague–Dawley rats, ovariectomy and MIA-induced OA)	Within rice porridge, 250 and 750 mg/kg, food supply was replaced every two days for 8 weeks	Prevention of bone loss	↑ BMD in lumbar bone spine, OA leg and control leg (DEXA) ↓ serum ALP activity (ELISA)	Yang et al.	(47)
**Oil extract of *Allium sativum L.* from raw cloves**	Rat model of OP (Female albinorats, ovariectomy)	Gavage, 100 mg/kg body wt/day, single evening dose for 30 days	Prevention of bone loss	↓ serum ALP activity (pNPP measurements) and TRAP activity (commercial kit) ↑ BMD of femur, thoracic rib, thoracic vertebra and lumbar vertebra (measured by Archimedes’ principle)	Mukherjee et al.	(90) (91)
				↑ calcium and phosphate content in femur, lumbar vertebra, thoracic vertebra, thoracic rib (method of Adeniyi et al. (1993) and Lowry and Lopez (1946))	Mukherjee et al.	(91) (92)
				↑ tensile strength of the femur (method of Shapiro and Heaney (2003) ↑ serum estradiol levels (ELISA) serum PTH levels is not affected (ELISA)	Mukherjee et al.	(92)
**Oil extract of *Allium sativum L.* from raw cloves**	Rat model of OP (Female Wistar, ovariectomy)	Gavage, 100 mg/kg body wt/day, single evening dose for 30 days	Increase in bone strength and inhibition of bone resorption	↑ tensile strength of the femurs (method of Shapiro and Heaney (2003) ↓ serum TRAP activity (commercial kit)	Mukherjee et al.	(93)
***Allium cepa L.* powder**	Rat model of OP (Female, 14-week-old, Wistar rats) treated or not with 1 mg/kg/day alendronate	Dietary administration, diet containing 3%, 7% and 14% (wt/wt) *Allium cepa L*. powder, for 6 weeks	Prevention of Ovx-induced bone loss and deterioration of biomechanical properties (efficacy was slightly inferior to that of alendronate)	↓ serum calcium (measured with an automatic chemistry analyzer) ↑ serum OCN (ELISA) ↑ BV/TV, Tb.N, ↓ Tb.Sp (histomorphometry on histological specimen) ↓ osteoclasts (TRAP staining on histological specimen) ↑ loading force to maximal load and tissue fracture, ↑ stiffness (three-point bending test)	Huang et al.	(94)
Diallyl disulfide (DADS) *	A mouse calvarial osteolysis model (Female, 6-wk-old, C57BL/6 mice, LPS treatment 5 mg/kg)	Subcutaneous injections, 20-40 mg/kg DADS, every alternate day for 14 days	Inhibition of LPS-induced osteolysis	↓ bone erosion as compared to LPS, ↑ BV/TV, ↓ porosity (microCT) ↓ osteoclasts (histologic and histomorphometric analysis TRAP staining)	Yang et al.	(86)
Allyl sulfide (AS) *	Age-associated OP mouse model (Female, 20-months-old (aged), C57BL/6 J mice)	Oral gavage, 200 mg/kg, 3-months	Restored osteogenesis and bone density	↑ plasma levels of P1NP and CTX-I ↑ bone density in the femur’s metaphyseal area (X-ray *in vivo* imaging)	Behera et al.	(83)
Allicin *	Mice model of lead-induced bone loss (Male, 3-weeks-old, C57BL/6 J mice, 0.2% lead acetate in drinking water ad libitum for 12 weeks)	Intraperitoneally injection, 10 mg/kg, in the last 4 weeks	Prevention lead-induced bone loss	↑ BMD, BVF, Tb.N, Tb.Th, ↓ Tb.Sp (microCT) ↑ CAT, SOD, reduced GSH; ↑ MDA on femur homogenates (commercial kits) ↓ TRAP, CTSK, NFATc1, MMP-9 mRNA in femur (RT-PCR) ↑ SIRT1 and ↓ of acetylated FOXO1 on femur homogenates (western blot)	Li et al.	(95)
Allicin *	Mice model of aging rats (Male, 13 months-old, F344 rats)	Intragastric administration, 4-8-16 mg/kg, once daily for 8 months	Reverse aging-associated bone loss and frailty	↑ femoral, spinal, tibial BMD (DEXA) ↑ elastic load and maximum load in femur - ↑ bone strength (Three-Point Bending Test) ↑ serum P1NP, ↑ serum CTX-I (ELISA)	Liu et al.	(96)

Most *in vivo* studies were conducted by using water or ethanol extracts of *Allium* edible plants (11 studies; *Allium macrostemon*, *Allium hookeri*, *Allium fistulosum*, *Allium sativum L.*, *Allium cepa L.*). A few studies used *Allium*-derived OSCs (4 studies; diallyl sulfide, allyl sulfide, allicin). Most studies were performed in normal control mice showing improved bone formation and inhibited bone resorption; and in osteoporosis mice showing prevention of bone loss. Notably, only the effects of purified OSCs (labeled with * in the table) can be attributable entirely to OSCs. Markers of bone formation in serum: procollagen 1 intact N-terminal propeptide (P1NP); osteocalcin (OCN); collagen I (Col I), alkaline phosphatase (ALP), parathormone (PTH). Markers of bone resorption in serum: serum type I collagen breakdown product (CTX-I). Markers of bone resorption in urine: urinary excretion of tritium. Bone microstructural parameters analyzed by microCT analysis: BMD (bone mineral density), bone volume fraction (BVF), spine BMD (s-SMD), tibia BMD (t-BMD), BMC (bone mineral content), bone volume (BV), bone volume/total volume (BV/TV), bone surface/bone volume (BS/BV), trabecular thickness (Tb.Th), trabecular number (Tb.N), trabecular space (Tb.Sp), bone volume fraction (BVF). Bone mineral density analyzed by dual-energy X-ray absorptiometry (DEXA). Markers of bone formation in histological specimen: ALP, Col I. Osteoid tissue detection by masson-goldner trichrome assay. Markers of osteoclasts/bone resorption in histological specimen: tartrate-resistant acid phosphatase (TRAP), nuclear factor of activated T-cells cytoplasmic 1 (NFATc1), cathepsin K (CTSK). Markers of redox stress response: catalase (CAT), superoxide dismutase (SOD), reduced glutathione (GSH), malondialdehyde (MDA). Measurements of bone strength: method of Shapiro and Heaney (2003); three-Point Bending Test. Other abbreviations: insulin-like growth factor 1 (IGF-1), bone morphogenetic protein 2 (BMP-2), lipopolysaccharide (LPS), sirtuin (SIRT); forkhead box O (FOXO). ↑ means up-regulation; ↓ means down-regulation.

**Table 4:** *Brassicaceae-*derived OSCs: effects on *in vitro* models of osteoclastogenesis and osteoblastogenesis.

Molecule (organosulfur compouds)	Experimental *in vitro* model	Concentration	Main effect	Specific outcomes	Authors	Ref
Sulforaphane *	MLO-Y4, an osteocyte – cell line	3-10-15-30-100 μM	Inhibits cells proliferation; induces apoptosis; and inhibits osteoclastogenesis	• ↓ viability and metabolic activity (3-(4,5-dimethylthiazol-2-yl)-2,5-diphenyltetrazolium bromide-like assay (EZ4U) • ↑ in the activities of Caspase 3/7 and 8 (assay kit) • ↑ Fas mRNA expression (RT-PCR) • ↓ RANKL mRNA expression (RT-PCR)	Thaler et al.	(97)
Glucoraphanin *	*In vitro* culture of human mesenchymal stromal cells from tibial plateau	3.3-10-33-100 μM	Induction of osteogenesis	• ↑ mineralization (alizarin red staining) • ↑ BSP, CBS, SMAD-1 mRNA (RT-PCR) • ↓ ALP, WISP-1 mRNA (RT-PCR)	Gambari et al.	(98)
***Brassica rapa* L. root ethanol extract**	MG-63 cells line	1-5-10-25-50 μg/ml	Increased osteogenesis	• ↑ viability (Wst-8 assay) • ↑ ALP activity (pNPP measurements) • ↑ collagen (Sirius Red) • ↑ mineralization (alizarin red staining)	Jeong et al.	(54)
Sulforaphane *	MC3T3-E1	3-10-15-20-30-100 μM SFN	Promotion osteoblast differentiation and induction of apoptosis	• ↓ cells proliferation (3-(EZ4U) • ↑ in the activities of Caspase 3/7 and 8 (assay kit) • ↑ Fas mRNA expression (RT-PCR) • ↑ mineralization (alizarin red staining) • ↑ RUNX-2 mRNA expression (RT-PCR)	Thaler et al.	(97)
Sulforaphane *	BMMSCs from long bones of 6-week-old C57BL/6 mice	3 μM	Promotes osteoblast differentiation	• ↑ mineralization (alizarin red staining) • ↑ RUNX-2 mRNA expression (RT-PCR)	Thaler et al.	(97)
**Hot water extract of *Brassica oleracea***	RAW 264.7 cell line	200 g/mL	Inhibition of osteoclast formation	↓ osteoclasts in femur, when in combination with P. ginseng extract (TRAP staining)	Kang et al.	(99)
Sulforaphane *	RAW 264.7 cell line	3-10-15-30-100 μM	Reduces proliferation and induces apoptosis	• ↓ viability and metabolic activity (EZ4U) • No alteration in Acp5, Clcr, and CTSK mRNA expression (RT-PCR) • ↑ Tet1 and Fas-Caspase 8-Caspase 3/7 pathway (western blot, assay kit)	Thaler et al.	(97)
Sulforaphane *	RAW 264.7 cell line	1-2-5-10 μM	Inhibition of osteoclastogenesis	• ↓osteoclasts (TRAP staining) • ↑ NRF2 protein accumulation (western blot); ↑ HO1, NQO1, GCLC and GCLM mRNA (RT-PCR) • ↓ ROS (2′,7′-Dichlorofluorescin diacetate) • ↓ NFATc1, C-FOS, TNFα, TRAP, CTSK, MMP-9, DC-STAMP mRNA (RT-PCR)	Xue et al.	(100)
Sulforaphane *	RAW 264.7 cell line	0.01-0.1-0.5-1 μM	1. Inhibits osteoclastogenesis 2. Inhibits osteoclasts cells-fusion	• induced cytotoxicity at > 5 μM (CCK-8 assay) • ↓ osteoclasts (TRAP assay) • ↓NFATc1, TRAP, CTSK mRNA (RT-PCR) • ↓ OSCAR, DC-STAMP, OC-STAMP mRNA (RT-PCR) • ↑ phosphorylation of STAT1 (Tyr701) (western blot)	Takagi et al.	(101)
Sulforaphane *	RAW 264.7 cell line	0.01-0.1-1-10 μM	Inhibition of osteoclastogenesis	• ↓ osteoclasts • ↓NF-kappaB activation	Kim et al.	(102)
Sulforaphane *	RAW 264.7 cell line	0.5, 1, 2.5, 5, 10, 20 μM	Decreased viability and osteoclastogenesis	• Marked cytotoxicity at concentration > 5 μM, low cytotoxicity 1-2.5 μM (CCK-8 assay) • ↓osteoclasts (TRAP staining) • ↓ CTSK, MMP-9 mRNA and protein (RT-PCR) • ↓ in autophagosomes and LC3-II, Beclin1, and Atg5–Atg12 mRNA and protein; ↓ of JNK phosphorylation (RT-PCR, western blot) • ↓size of F-actin rings	Luo et al.	(103)
Sulforaphane *	Primary mouse osteoclasts from tibial and femoral bone marrow of 8-week-old C57BL/6 mice	3 μM	Inhibition of osteoclasts resorption	↓ resorption activity	Thaler et al.	(97)
Sulforaphane *	Primary osteoclast precursors isolated from BM of tibias and femurs of 8–12 weeks old male C57BL/6 mice	1-5 μM	Inhibition of osteoclastogenesis	↓ osteoclasts (TRAP staining)	Xue et al.	(100)
Sulforaphane *	BM cells obtained from the femur and tibia of 7–10-week-old ddY male mice	0.01-0.1-0.5-1 μM	Inhibition of osteoclastogenesis	• induced cytotoxicity at > 5 μM (CCK-8 assay) • ↓ osteoclasts (TRAP staining) • ↓ NFATc1, TRAP, CTSK mRNA expression (RT-PCR)	Takagi et al.	(101)
Sulforaphane *	BM cells isolated from femora and tibiae of 4- 6-week-old C57BL/6 mice	0.01-0.1-1-10 μM	Inhibition of osteoclastogenesis	• ↓ osteoclasts • Early inhibition of osteoclastogenesis • No effects on osteoclasts resorption • No effects on RANK or c-fms mRNA	Kim et al.	(102)
Sulforaphane *	BMMs from 5-week-old C57BL/6 female mice	1, 2.5, 5 μM	Decreased viability and inhibition of osteoclastogenesis	• Moderate cytotoxicity at concentration >2.5 μM (CCK-8 assay) • ↓ osteoclasts (TRAP staining)	Luo et al.	(103)
Sulforaphane *	Human monocytes isolated from peripheral blood of healthy volunteers	0.2-1-5 μM	Inhibition of osteoclastogenesis	• ↓ osteoclasts (TRAP staining) • ↑NRF2 accumulation (immunocytochemistry) • ↑ NQO1 and PRDX1 mRNA expression (RT-PCR)	Gambari et al.	(104)

Most in vitro studies were conducted using purified OSCs (6 studies, 15 in vitro models; sulforaphane, glucoraphanin); while only a few used water or ethanol extracts from Brassicaceae edible plants (2 studies, 2 in vitro models; Brassica rapa, Brassica oleracea). Most studies showed increased osteogenesis and decreased osteoclastogenesis. Notably, only the effects of purified OSCs (labeled with * in the table) can be attributable to OSCs. The concentrations tested ranged from 0.01 to 100 μg/ml. Murine in vitro models of osteoclastogenesis: osteoclasts derived from bone marrow of femora and tibiae of mice, RAW 264.7 cell line. Human in vitro models of osteoclastogenesis: human monocytes isolated from peripheral blood of healthy volunteers. Murine in vitro models of osteoblastogenesis: MC3T3-E1 (Mouse C57BL/6 calvaria cells line); murine bone marrow (BM) cells; bone marrow-derived mesenchymal stem cells (BMMSCs), bone marrow macrophages (BMMs). Human in vitro models of osteoblastogenesis: MC3T3-E1, MSCs isolated from human tibial plateau. Osteocyte – cell line: MLO-Y4. Functional assays for osteoclastogenesis: tartrate-resistant acid phosphatase positive (TRAP staining); pit assay. Functional assays for osteoblastogenesis: Alizarin red staining (marker of mineralization), Sirius red assay (marker of collagen I), p-nitrophenyl phosphate (pNPP) quantification. Proliferation/viability assays: cell counting kit-8 (CCK-8) cell viability assay, water-soluble tetrazolium-8 (WST-8) assay, 3-(4,5-Dimethylthiazol-2-yl)-2,5-diphenyltetrazolium bromide-like assay (EZ4U). Markers of osteoclasts: nuclear factor of activated T-cells cytoplasmic 1 (NFATc1), cathepsin K (CTSK), receptor activator of NF-KB (RANK), osteoclast stimulatory transmembrane protein (OC-STAMP), tartrate-resistant acid phosphatase (TRAP), receptor activator of nuclear factor-κB ligand (RANKL), dendritic cell specific transmembrane protein (DC-STAMP), reactive oxygen species (ROS), c-fos, nuclear factor kappa-light-chain-enhancer of activated B cells (NF-κB), matrix metallopeptidase 9 (MMP-9), osteoclasts-specific activating receptor (OSCAR), acid phosphatase 5, tartrate resistant (ACP5), calcitonin receptor-like receptor (Clcr), colony-stimulating factor-1 receptor (c-fsm), c-fos. Markers of osteoblastogenesis: cystathionine-β-synthase (CBS), bone sialoprotein (BSP), SMAD family member 1 (SMAD-1), alkaline phosphatase (ALP), WNT1-inducible-signaling pathway protein 1 (WISP-1), osteocalcin (OCN), runt-related transcription factor 2 (RUNX-2). Markers of cell viability – apoptosis: Fas, Caspase 3/7 and 8, nuclear factor erythroid-derived 2-related factor 2 (NRF2), heme oxygenase-1 (HO1), NAD(P)H: quinone oxidoreductase 1 (NQO1), peroxiredoxin-1 (PRDX-1), glutamate cysteine ligase catalytic subunit (GCLC), glutamate-cysteine ligase modifier subunit (GCLM), peroxiredoxin 1 (PRDX-1), microtubule-associated protein 1A/1B-light chain 3 (LC3-II), beclin1, autophagy related 5 (ATG5), Jun N-terminal kinases (JNK), autophagy related 12 (Atg12). ↑ means up-regulation; ↓ means down-regulation

**Table 5:** *Brassicaceae-*derived OSCs: effects on *in vivo* models of bone loss.

Molecule tested	Experimental *in vivo* model description	Mode of administration, dose and duration	Main effect	Specific features	Authors	Ref
**Sulforaphane**	C57BL/6 mice, Mouse calvarial models treated with LPS (10 mg/kg body weight injected in calvaria)	Intraperitoneal injection, 10 mg/kg body weight, the day before LPS treatment for 6 days	Protection against LPS-induced calvarial bone erosion by inhibition of osteoclastogenesis	• ↑ BV/TV, Tb.N, ↓Tb.Sp (microCT) • ↓ osteoclasts (TRAP staining in histological samples) • ↓ CTSK (immunohistochemical and immunofluorescence analysis)	Luo et al.	(103)
**Sulforaphane**	*Ex vivo* culture of calvariae explants of 2–3-day-old and 7-week-old, C57BL/6 mice	3 μM	Promotes osteogenesis inhibits osteoclastogenesis	• ↑ ECM mineralization (alizarin red staining on calvaria tissue) • ↓ RANKL (RT-PCR on calvariae lysates)	Thaler et al.	(97)
**Sulforaphane**	Mice model of OP (Female, 8-week-old, C57BL/6 mice, ovariectomy)	Intraperitoneal injection, 7.5 mM DL-SFN, every other day for 5 weeks	Prevention of bone loss	• ↑ BV/TV, Tb.N ↓Tb.Sp, no effect on Tb.Th or Co.Th in tibiae (micro CT)	Thaler et al.	(97)
**SFX- 01^®^ (a stable form of Sulforaphane)**	Osteoarthritis model (Male, 26-week-old, STR/Ortmice)	Oral administration, 100 mg/kg, daily for 3 months	Improvements in cortical bone mass	• ↑ TV, BV and BV/TV of tibial epiphyseal trabecular bone and metaphyseal trabecular bone (micro CT) • ↑serum P1NP (ELISA) • ↓serum CTX-I (ELISA)	Javaheri et al.	(105)
***Brassica rapa L.* root ethanol extract**	Female, 3-week-old, Sprague- Dawley rats	Oral administration, 500 mg/kg/day, single daily dose for 6 weeks	Increased bone formation	• ↑ BMD, BV, BV/TV, Tb.N, Tb.Th., ↓Tb.Sp. (microCT) • ↑ serum OCN (immunoassay)	Jeong et al.	(54)
***Lepidum sativum* seed extract**	Rat model of OP (Female Wistar rats, ovariectomy)	Oral gavage 50 and 100 mg/kg	Prevention of bone loss and bone strengthening activity	• ↑ femur weight (weights were calculated as wet femur weight/body weight) • ↑ femur compression strength (hardness tester (Erweka GmbH, Heusen-stamm, Germany) • ↑ ALP, OCN serum levels; ↓ TRAP, CTX-I serum levels (ELISA) • ↓ RANKL, ↑ OPG mRNA (RT-PCR)	Abdallah et al.	(59)
***Lepidum sativum* seed**	Glucocorticoid-induced OP (GIO) model (Female Wistar rat, subcutaneous injection of methylprednisolone 3.5 mg/kg per day for 4 weeks)	Oral gavage, 6 g of LS seeds in diet daily	Prevention of GIO-dependent bone loss	• ↑ percentage of trabecular bone *vs* GIO (histopathological examination and Image J quantification) • ↓ serum TRAP *vs* GIO (commercial kit) • ↑ serum b-ALP (immunoassay), phosforous and calcium (automated analyser) *vs* GIO	Elshal et al.	(106)
***Lepidum* sativum seed**	Fracture-induced healing model (New Zealand White rabbits, induced fractures in the midshaft of the left femur)	Oral gavage, 6 g of *Lepidum sativum* seeds in their food daily after surgery	Increased healing of fractures	Increased callus formation in fractures (x-rays and quantification)	Juma et al.	(83)
**Methanolic and aqueous extract of *Lepidium sativum* seeds**	Fracture healing model (Charles foster rats, hand held three-point bending technique)	Oral administration, methanolic extracts 400 mg/kg or aqueous extracts 550 mg/kg, from the day of fracture induction for 2 months	Increased healing of fractures	• Larger callus formation (x-rays and quantification) • ↑ calcium, phosphorus, and ALP serum levels (commercial kits)	Dixit Jr Iii et al.	(28)
***Lepidium sativum* seeds**	Glucocorticoid-induced OP (Adult male guinea pigs, methyl prednisolone 3.5 mg/kg per day for 4 weeks subcutaneously)	Oral administration trough a gastric tube, 300 mg/kg, for 4 weeks	Prevention of bone loss in femur	• Prevention of caspase-3 activation (caspase-3 immunostaining) • Prevention of decrease of OPN (immunohystochemistry) • Prevention of decrease in osteoblast and Co.th. in femur (histomorphometric analysis) • Prevention of increase of osteoclasts in femur (histomorphometric analysis)	EL-Haroun et al.	(107)
**Ethanol extracts of Maca root (*Lepidium meyenii Walp*.)**	Rat model of OP (Female, 90-day-old, Sprague-Dawley rats, ovariectomy)	Oral gavage, 0.096 and 0.24 g/kg, for 28 weeks	Prevention of estrogen deficient bone loss	• ↑ calcium content of femur (Atomic Absorption Spectrophotometer) • ↑ BMD and trabecular bone of the lumbar vertebrae (DEXA) • ↑ serum OCN (radioimmunoassay commercial kit)	Zhang et al.	(108)
**Hot water extract of *Brassica oleracea* (Bo)**	Mice model of OP (Female, 7-week-old, C57BL/6 mice, ovariectomy)	Oral administration, 500 mg/kg, daily for 10 weeks	Inhibits OVX-induced bone loss	• ↑ BMD when in combination with Panax ginseng (DEXA) • ↓ osteoclast number when in combination with Panax ginseng (immunohistochemistry, TRAP staining)	Kang et al.	(99)

Most *in vivo* studies were conducted by using water or ethanol extracts of *Brassica* edible plants (8 studies; *Brassica rapa, Lepidum sativum, Lepidum meyenii Walp, Brassica oleracea*). A minority of studies used *Brassicaceae*-purified OSCs (3 studies; 4 models; SFN, SFX-01). Most studies were performed in osteoporosis mice showing prevention of bone loss. Notably, only the effects of purified OSCs (labeled with * in the table) can be attributable entirely to OSCs. The route of administration was mainly by oral administration. Markers of bone formation in serum: procollagen 1 intact N-terminal propeptide (P1NP); osteocalcin (OCN). Markers of bone resorption in serum: serum type I collagen breakdown product (CTX-I), tartrate-resistant acid phosphatase (TRAP), osteoprotegerin (OPG), cortical thickness (Co.Th). Bone microstructural parameters analyzed by microCT analysis: BMD (bone mineral density), bone volume (BV), bone volume/total volume (BV/TV), trabecular thickness (Tb.Th), trabecular number (Tb.N), trabecular space (Tb.Sp.). Bone mineral density analyzed by Dual-energy X-ray absorptiometry (DEXA). Markers of bone formation in histological specimen: alkaline phosphatase (ALP), osteopontin (OPN). Markers of osteoclasts/bone resorption in histological specimen: tartrate-resistant acid phosphatase (TRAP), cathepsin K (CTSK). Measurements of bone strength: Erweka GmbH, Heusen-stamm Germany. Extracellular matrix (ECM). Markers of osteoclast in histological specimen: receptor activation of nuclear factor-kB ligand (RANKL). ↑ means up-regulation; ↓ means down-regulation. ↑ means up-regulation; ↓ means down-regulation.

Importantly, while data obtained from studies on purified molecules (labeled with * in the tables) clearly attest to the effectiveness of individual OSCs, the effect of OSCs-rich extracts may result from the combined action of other phytochemicals contained in the extracts. Indeed, *Allium* species contains polyphenols, flavonoids, flavanols, anthocyanins, tannins, ascorbic acid, saponins and fructans (109–111); *Brassica* species contains ascorbic acid, phenolics, carotenoids, terpenes, phytoalexins and alkaloids (29, 112).

### Regulation of osteogenesis and bone formation

Osteoblasts, the bone forming cells, regulate bone homeostasis by synthesizing a wide variety of extracellular protein of bone matrix. They differentiate from MSCs through the osteogenic differentiation process which is regulated by an orchestrated activation of several pathways. The master regulator of osteogenic differentiation is runt-related transcription factor 2 (RUNX-2), which is expressed in the early stages of differentiation and is at the intersection of several signaling pathways among which growth hormone-janus Kinase 2 (GH-JAK2), bone morphogenetic protein-SMAD (BMP-SMAD), canonical Wingless/Integrated (Wnt) and Notch signaling (113, 114). Among the genes targeted by RUNX-2 are osteocalcin (OCN), collagen I (Col I), bone sialoprotein (BSP), osteopontin (OPN), alkaline phosphatase (ALP). BSP, OPN and ALP are correlated to matrix mineralization; Coll I and OCN are among the major components of bone matrix. Wnts-β-catenin signal activates osteogenic target genes such as distal-less homeobox 5 (Dlx5) and osterix (Osx) (115) and suppresses the transcription of adipogenic transcription factors such as peroxisome proliferator-activated receptor-γ (PPAR-γ) (116). SMAD family number 1 (SMAD-1) is a critical immediate downstream mediator of BMP receptor transduction (117). Among downstream targets of canonical Wnt and BMP signaling is WNT1-inducible signaling pathway protein 1 (WISP-1), which is involved in the positive regulation of osteogenesis and negative regulation of adipogenesis (118). Interestingly, the expression of H_2_S generating enzymes, cystathionine-β-synthase (CBS) and cystathionine-γ-lyase (CSE), was found to be transcriptionally up-regulated during osteogenesis and to correlate with the biosynthesis of mineral matrix (119), thus suggesting a role for endogenous H_2_S in osteogenic differentiation. Osteogenic differentiation is associated to increased ALP activity and mineralization *in vitro* and increased BMD *in vivo*. Osteoblast finally differentiate toward osteocytes, multifunctional bone cells that are embedded in mineralized bone matrix. Osteocytes act as orchestrators of bone remodeling, through regulation of both osteoclast and osteoblast activity; as regulators of phosphate metabolism and calcium availability, by functioning as an endocrine cell; as mechanosensory cells (120). Key factors produced by osteocytes are sclerostin (a negative regulator of bone mass), FGF-23 (a regulator of phosphate metabolism), and the key regulator of osteoclast differentiation receptor activator of nuclear factor κβ ligand (RANKL), also produced by osteoblasts and MSCs (120, 121).

Most studies investigating OSCs extracts focused on a commonly used human osteoblastic model, the human osteosarcoma cell line (MG-63 cells). They showed increased cell proliferation and increased osteogenesis/mineralization by *Allium Hookeri* roots treatments (48); increased osteogenesis by *Allium fistulosum* (80) and *Brassica Rapa L.* (Jeong); while no effect on proliferation and differentiation was shown by treatment with water solution of onion crude powder (81). However, MG-63 cells are osteoblasts derived from osteosarcoma, a malignant bone tumors, thus are not fully representative of physiological osteoblasts (122). Increased cells proliferation by *Allium* genus was also shown by ginger and garlic extracts released by 3D-printed calcium phosphate scaffolds on human fetal osteoblast cells (82); increased osteogenesis by *Allium fistulosum* was also shown in the mouse C57BL/6 osteoblastic calvaria cell line (MC3T3-E1) (80). Up to date no studies on primary cultures of human MSCs have been performed with extracts derived from *Alliaceae* or *Brassicaceae*.

Treatment with *Alliaceae* extracts improved bone formation in normal control rats (41, 48, 88) and mitigated the bone loss due to several pathological conditions among which osteoporosis (47, 80, 94). Similarly, extracts from *Brassicaceae* induced bone formation in control rats (54) and prevented bone loss in several models of osteoporosis (59, 99, 106–108). Interestingly, treatment with *Lepidium sativum* resulted in improved fracture healing (28, 123).

Notably, several studies focused on purified OSCs molecules, revealing a specific effect of OSCs on proliferation, osteogenic differentiation, and bone formation. Behera et al. showed increased proliferation, ALP activity and mineralization in murine MSCs derived from femur bone marrow (BMMSCs) upon allyl sulfide stimulation, with a mechanism implicating increased RUNX-2 and OCN expression (83). Thaler et al. demonstrated increased mineralization in mouse MSCs and in an *ex vivo* culture of calvariae explants treated with SFN (97); at the molecular level, SFN induced up-regulation of RUNX-2 in mouse MSCs (97). Gambari et al. showed increased mineralization and BSP, CBS and SMAD-1 mRNA up-regulation by GRA administration in primary human MSCs (98). Finally, with regards to osteocyte regulation, Thaler et al. showed that SFN inhibited proliferation in murine osteocyte-like cell line (MLO-Y4) (97).

Purified OSCs have also been tested in *in vivo* models of bone loss or osteolysis, showing beneficial effects on preserving bone mass. Oral administration of allyl sulfide in an age-associated osteoporosis mouse model resulted in increased bone density at X-ray analysis and increased serum levels of procollagen 1 intact N-terminal propeptide (P1NP; a marker of bone formation) (83). Similarly, intragastric administration of allicin increased BMD, as detected by dual energy X-ray absorptiometry, and bone strength, as measured by three-point bending assay, in a model of aging osteoporotic rats (96). Intraperitoneal administration of allicin prevented the bone loss in a mice model of lead-induced bone loss (osteoporosis induced by a toxic heavy metal), as measured by increased BMD, trabecular number (Tb.N), trabecular thickness (Tb.Th) and decreased trabecular space (Tb.Sp), quantified using micro-CT analysis (95). Finally, SFN showed to be protective against bone loss in different *in vivo* models. Intraperitoneal injection of SFN in lipopolysaccharide (LPS)-induced erosion of the mice calvaria bone induced increased trabecular bone volume (BV/TV), increased Tb.N and decreased Tb.Sp, as measured by micro-CT analysis (103); moreover, intraperitoneal injection of SFN in a mice model of ovariectomy-induced bone loss stimulated trabecular bone formation, increased Tb.N and decreased Tb.Sp (97); finally, the oral administration of SFN-01 (a stabilized form of SFN) in a mice model of osteoarthritis, resulted in increased trabecular bone volume and serum P1NP (105).

### Regulation of osteoclastogenesis and bone resorption

Osteoclasts are bone-resorbing cells which arise from immature monocytes and mature tissue macrophages (124). Osteoclasts differentiation stems from the signaling triggered by two critical cytokines produced by MSCs, osteoblasts and osteocytes: macrophage colony-stimulating factor (M-CSF) and RANKL binding, respectively, to the receptors colony-stimulating factor-1 receptor (c-fms) and receptor activator of nuclear factor κ B (RANK) (125, 126). RANKL signaling activation induces various intracellular signal transduction cascades such as tumor necrosis factor receptor-associated factor 6 (TRAF-6), NADPH oxidase 1 (NOX-1), RAC family small GTPase 1 (RAC1), nuclear factor kappa-light-chain-enhancer of activated B cells (NF-κB), and nuclear factor-activated T cells c1 (NFATc1), c-fos (127–129). Other receptors involved in osteoclastogenesis are calcitonin receptor (CTR), ITAM bearing Fc receptor standard g chain (FcRγ), osteoclasts-specific activating receptor (OSCAR) (126, 130); key signaling is mediated by mitogen-activated protein kinases (MAPK), and includes extracellular signal-regulated kinase (ERK), c-Jun N-terminal kinase (JNK), and p38 activation. Moreover, critical to osteoclast differentiation and function are: intracellular reactive oxygen species (ROS) generation, which act as key signaling molecules (82, 88, 94); osteoclast fusion mediated among other factors, by the fusogenic molecules osteoclasts-stimulatory transmembrane protein (OC-STAMP) and dendritic cell-specific transmembrane protein (DC-STAMP) (126, 131, 132); and expression of specific enzymes such as tartrate-resistant acid phosphatase (TRAP), cathepsin K (CTSK) (126, 130), tartrate-resistant acid phosphatase 5b (TRAP5b) (83) and matrix metallopeptidase 9 (MMP-9).

Extracts from both *Allium* and *Brassica* species were shown to attenuate osteoclast differentiation *in vitro* in the murine macrophage cell line, RAW 264.7. In particular, extracts of onion (85), freeze dried onion juice (85), solution of onion crude powder (81) inhibited osteoclastogenesis, as measured by TRAP staining *in vitro*. A similar effect was achieved by an extract of *Brassica oleracea* but only in combination with extract from *Panax ginseng* (99). Using human THP1 monocytes, Bose et al. showed that ginger and garlic extracts reduce the frequency and the size of resorption pits carved by osteoclasts (82); inhibition of osteoclast number was found also by onion and commercial onion extracts in rat and rabbit osteoclasts (81). Notably, Wetli et al. demonstrated that onion extract reduced rat osteoclast differentiation and were able to isolate a specific sulfoxide component of onion powder, γ-glutamyl-*trans*-*S*-1-propenyl-l-cysteine sulfoxide (GPCS), which the authors found to be the key responsible of this biological activity (41).

*In vivo* administration of extracts rich in OSCs decreased osteoclastogenesis and bone erosion in rodent model of osteoporosis; Huang et al. showed that ovariectomized rats fed with different concentrations of onion extracts (up to 14% wt/wt in the diet powder) were partly protected against loss of bone mass and bone material properties (94); moreover, histomorphometry revealed that treatment with onion extracts was associated with a lower number of osteoclasts *in vivo* (94). Similar findings were reported by Kang et al. using ovariectomized mice fed with a combination of extracts obtained from *Panax ginseng* and *Brassica oleracea* (99). Furthermore, Abdallah HM et al. reported that ovariectomized rats treated with extracts of *Lepidium sativum* were partly protected against osteoporosis and showed a sharply decreased RANKL/osteoprotegerin (OPG) ratio in femur bones (59).

Studies that used purified OSCs molecules further supported efficacy and specificity. Yang et al. demonstrated a dose-dependent inhibition of osteoclast differentiation and a decreased bone resorption by mature osteoclasts upon treatment with DADS (86). Monocytes proliferation and viability was inhibited by SFN (97).

Luo et al. (103) and Xue et al. (100) showed that SFN inhibits osteoclast differentiation in RAW 264.7 murine macrophagic cell line; Takagi et al. (101) and Kim et al. (102) showed similar findings in murine BM cells and so did Gambari et al. (104) in a model of osteoclast derived from human monocytes. Moreover, Chen et al. reported the inhibition of osteoclast differentiation by alliin in RAW 264.7 *via* scavenging of ROS signaling (87).

Mechanisms of regulation of osteoclastic differentiation by OSCs involved different molecular targets. Li et al. reported that the anti-osteoclastogenic activity of allicin in mice is associated to the activation of the SIRT1/FOXO1 pathway and ROS scavenging (95). Similarly, one key mechanism of action of SFN is the activation of the master regulator of the antioxidant defense system, nuclear factor erythroid-derived 2-related factor 2 (NRF2), and its downstream target antioxidant and detoxifying enzymes (133), which is known to actively inhibit mouse osteoclasts differentiation *in vitro* (104, 134). SFN modifies sulfhydryl groups in kelch-like erythroid-cell-derived protein with CNC homology (ECH)-associated protein (KEAP-1), causing KEAP-1 dislocation, NRF2 stabilization and nuclear translocation (135); moreover, SFN regulates NRF2 expression *via* epigenetic mechanisms (136). Coherently, SFN was shown to increase NRF2 protein accumulation in RAW 264.7 murine cell line, to increase the expression of some NRF2-mediated antioxidant genes (heme oxygenase-1, HO1; NAD(P)H: quinone oxidoreductase 1, NQO1; glutamate cysteine ligase catalytic subunit, GCLC; ligase modifier subunit, GCLM) and decrease intracellular ROS production, and the overall number of osteoclasts as shown by Xue et al. (100). Similarly, SFN was shown to inhibit the osteoclast differentiation of human monocytes while increasing NRF2 nuclear translocation and protein expression of NRF2-mediated antioxidant genes (NQO1; Peroxiredoxin 1, PRDX-1), as published by Gambari et al. (104). Finally, SFN induces Caspase 8 and 3/7, thus inducing apoptosis in a RAW 264.7 murine cell line as shown by Thaler et al. (97).

Moreover, downregulation of the key transcription factor NFATc1 is implicated in several studies showing inhibition of osteoclast development: Yang et al. reported a dose-dependent down-regulation of NFATc1 in a RAW 264.7 murine cell line after DADS treatment (86); Xue et al. (100) and Takagi et al. (101), respectively, reported similar findings in RAW 264.7 murine cell line and in murine BM cells after SFN treatment; Behera et al. in murine BM cells after allyl sulfide treatment (83). The inhibition of other key transcription factor c-Fos and Nf-kB was shown by Yang et al. in a RAW 264.7 murine cell line after DADS treatment (86).

Several other proteins implicated in the adhesion an (83, 95, 100, 137), in RAW264.7 cells and murine BM and are detailed in **Tables 2**, **4**.

OSCs can modulate the expression of osteoclasts-specific activating receptors, necessary for the co-stimulatory signaling with immunoreceptors and prevented osteoclast fusion by inhibiting fusogenic molecules. Takagi et al. showed in RAW 264.7 murine cell line that OSCAR is inhibited by SFN (101). DC-STAMP was found inhibited in RAW 264.7 murine cell line after SFN treatment as shown by Takagi et al. (101) and by Xue et al. (100) and after DADS treatment as shown by Yang et al. (86). OC-STAMP was found inhibited in RAW 264.7 murine cell line after SFN treatment as shown by Takagi et al. (101).

Finally, OSCs compounds were shown to inhibit osteoclast differentiation *via* a paracrine mechanism, acting on osteoclasts-supporting cells. Thaler et al. showed that RANKL was inhibited by SFN in a murine osteocytes cell line (MLO-Y4) (97). Behera et al. showed that RANKL was inhibited while OPG was increased in supernatants of murine MSCs cells culture treated with allyl sulfide (83); and that treatment with this conditioned medium inhibited the expression of RANK and osteoclast differentiation of murine bone marrow (BM) cells (83).

Only a few *in vivo* studies used purified OSCs to investigate bone metabolism. In a mice model of lead-induced bone loss, intraperitoneal injection of allicin alleviates bone loss by preventing oxidative stress and osteoclastogenesis by modulating SIRT1/FOXO1 pathway (95). SFN treatment in a mouse calvaria model treated with LPS decreased the number of osteoclasts (103). Treatment of *Lepidium sativum* in a rat model of ovariectomy-induced osteoporosis improved mechanical properties of femurs while decreasing TRAP, serum type I collagen breakdown product (CTX-I), RANKL (59) and the number of osteoclasts (107).

## H_2_S release from OSCs as a potential mechanism of bioactivity in bone

H_2_S is a pleiotropic molecule which provides numerous health benefits by improving hypertension and cardiometabolic disorders (138) (139), relieving pain (140, 141), and increasing insulin sensitivity (142); protecting against neurological diseases including Alzheimer disease (143). Moreover, H_2_S is critically involved in the extension of lifespan provided by caloric restriction (144, 145). Supraphysiological levels of H_2_S may be generated in certain pathological conditions and lead to toxicity, inducing inflammation or tissue damage (146).

The intriguing overlap between biological effects attributed to some *Allium* and *Brassica* species and those exhibited by the gasotransmitter H_2_S prompted several researchers to verify the H_2_S releasing capacity of those molecules. Recently, the ability of releasing H_2_S was found as a distinctive feature of several OSCs, and a plausible mechanism for their biological effects across different organs and tissues was described. The biological relevance of H_2_S release by OSCs was first demonstrated by Benavides et al. in the context of a study on the vasoactivity of garlic. The authors showed that garlic polysulfides DATS and DADS, the downstream metabolites of alliin, released H_2_S in red blood cells; importantly, pre-treating the cells with the thiol-blocking reagent iodoacetamide inhibited the release of H_2_S, thereby demonstrating that the mechanism by which polysulfides release H_2_S is dependent on intracellular thiols, such as glutathione (GSH) (147). Chemically, this reaction involves a nucleophilic substitution from thiol at the α carbon of the H_2_S-donor moiety and a subsequent release of H_2_S (148). This mechanism is biologically relevant as the relaxation induced by both garlic extract and DADS on isolated rat aortic rings strongly correlated to the amount of H_2_S released. In the wake of this work, Citi et al. first revealed that a similar mechanism accounts for the ability of several *Brassicaceae*-derived ITCs to release pharmacologically relevant concentrations of H_2_S in an l-cysteine dependent manner (149): allyl isothiocyanate (AITC), 4-hydroxybenzyl isothiocyanate (HBITC), benzyl isothiocyanate (BITC), erucin (ER), SFN (149, 150). The same group reported that H_2_S-release is associated with the *in vivo* anti-hypertensive, hypoglycemic, pain-relieving, and anti-inflammatory effects of OSCs derived from the *Brassicacea Eruca Sativa* (138, 151–153). Interestingly, Lucarini et al. first demonstrated that GRA, a GLS, can release H_2_S in aqueous solution independent of myrosinase, but the chemical mechanism underlying this phenomenon is still unclear (150). Whether other *Alliaceae* or *Brassicaceae*-derived OSCs releases H_2_S is still unknown.

**Figure 3:**
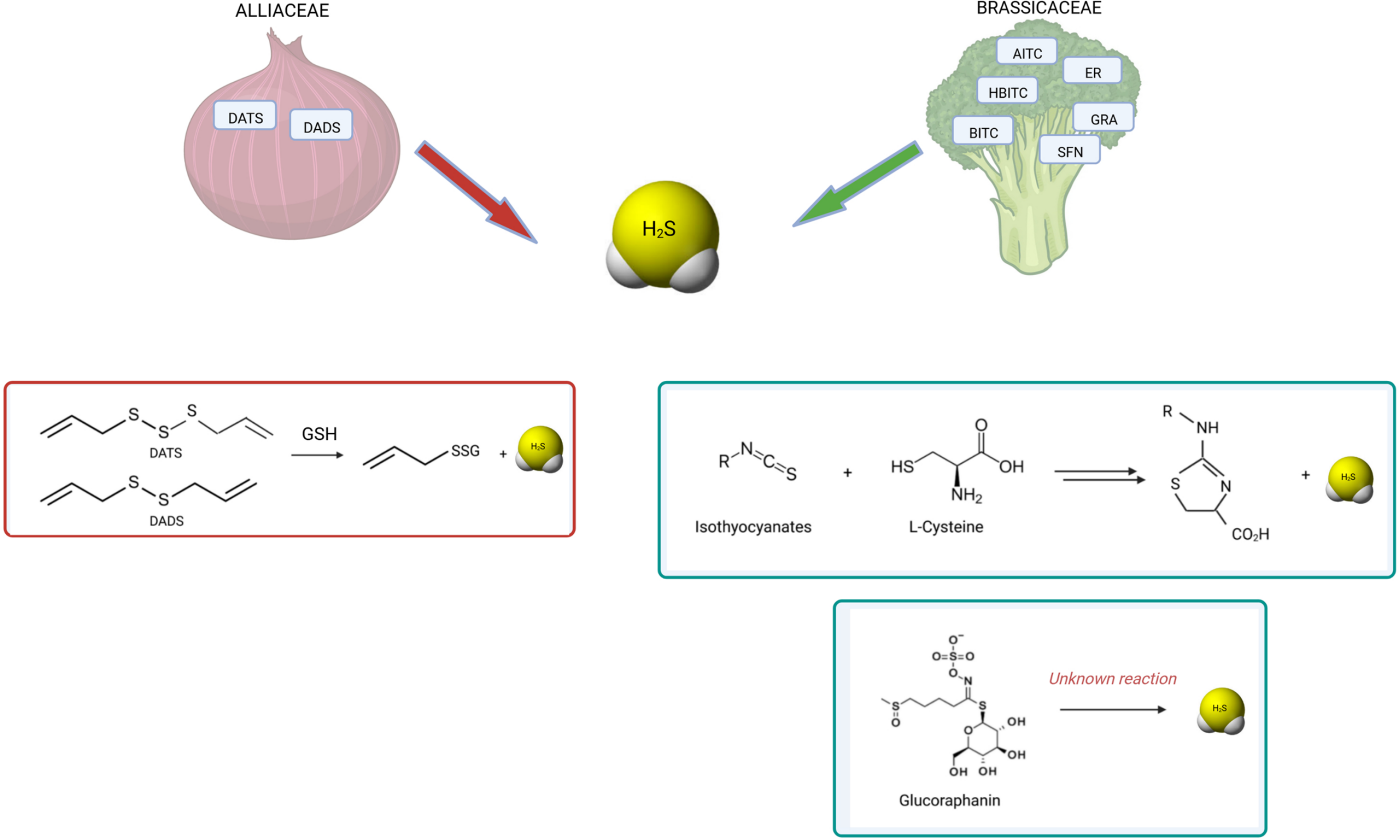
H_2_S release by OSCs derived from *Alliaceae* and *Brassicaceae*. The known reactions occurring for H_2_S release by polysulfides and isothiocyanates are shown. Among garlic-derived polysulfides, diallyl disulfide (DADS) and diallyl trisulfide (DATS) have been shown to release H_2_S by reaction with glutathione (GSH) by polarographic H_2_S sensor (154) (147) (148). Among glucosinolates, GRA has been found to release H_2_S by amperometric approach (149). Similarly, several isothiocyanates showed H_2_S-releasing activity: allyl isothiocyanate (AITC), 4-hydroxybenzyl isothiocyanate (HBITC), benzyl isothiocyanate (BITC), erucin (ER), sulforaphane (SFN) (149) (150). While the mechanism of release is unknown for glucosinolates, the mechanism of release by isothiocyanates is dependent on L-cysteine reaction (155). Moreover, different OSCs have different kinetics of H_2_S release.

**Figure 3** summarizes the known reactions leading to H_2_S release from polysulfides, GLS or ITCs.

This mechanism holds important implications for bone. Recent findings by our group and others demonstrated that H_2_S plays an important role in the regulation of bone cell differentiation and function. *In vitro*, H_2_S-donors promote osteogenic differentiation and stimulate mineralization by increasing calcium intake (156) and the expression of genes directly involved in the biosynthesis of hydroxyapatite, such BSP (157). Furthermore, the expression of the enzymes CBS and CSE, which are responsible for endogenous H_2_S production, steadily increased during osteogenic differentiation and correlated to mineral apposition (119). Moreover, H_2_S-donors inhibit osteoclast maturation and resorption by activating the antioxidant response elicited by the NRF2 transcription factor (104, 158). Further attesting to the relevance of H_2_S in bone homeostasis, evidence from several *in vivo* preclinical models showed that the depletion of H_2_S levels is associated with loss of bone mass; similar findings were reported in ovariectomized mice (157), in H_2_S-deficient CBS+/− mice (156), in glucocorticoids-induced osteoporosis (159). Interestingly, when animals were treated with pharmacological H_2_S-donors to normalize the plasma level of H_2_S, bone loss was prevented or reversed (156, 157). The ability of H_2_S to stimulate bone formation appears to be maintained across various conditions, even unrelated to systemic or genetic disfunctions: for example, the exogenous administration of H_2_S by means of the pharmacological donor GYY4137 was effective to attenuate the bone loss induced by modelled microgravity (160) and to promote osteogenesis in a model of distraction osteogenesis (161).

Overall, these data demonstrate that H_2_S regulates osteogenesis and bone formation in both healthy and pathological conditions.

Therefore, H_2_S release by OSCs could account, at least in part, for their biological properties. However, up to date no clinical or preclinical *in vivo* studies have investigated the effect of OSCs by correlating their bioactivity to the H_2_S levels.

## The GRA/SFN system: A case-model for OSCs bioactivity based on H_2_S release

GRA is a glucosinolate abundant in aerial portions, developing florets (flower buds), sprouts, seeds and mature plants of cabbage, broccoli, cauliflower, kale and Brussels sprouts (77). GRA conversion to SFN, an ITC, requires the enzyme myrosinase, an intracellular thioglucosidase, which catalyzes its hydrolysis to an unstable aglucone that spontaneously rearranges to give rise to a range of products, including SFN. SFN is the progenitor of a family of compounds widely studied in the literature mostly due to their antioxidant and anticancer properties. In mammalians, GRA conversion to SFN is primarily mediated by bacterial microflora of the gastrointestinal tract; while a small proportion is generated in the mouth by plant myrosinase when released by plants after chewing. Our current knowledge on the bioavailability and the rate of conversion of GSL into ITCs are largely based on studies on the GRA/SFN system.

Although most of GRA introduced with diet undergoes hydrolysis in the gut by microbial thioglucosidases, a fraction of GRA (around 10-15%) is absorbed directly in the stomach and in the small intestine, before the catabolic breakdown to SFN is triggered by gut microbiota (77, 162).

Gastric acidity appears to attenuate GSL bioavailability (163). However, GRA is not destroyed by digestive enzymes during passage through the digestive tract and is able to reach the rat cecum intact, when is hydrolyzed to SFN which is able to cross the cecal enterocyte for systemic absorption and enterohepatic circulation (164, 165). Conversion of GRA to bioactive SFN by the rat cecal microbiota requires four or more days after broccoli consumption and is reversible (166); however, recent randomized clinical trials have ascertained that upon ingestion of GRA-enriched soups, increased SFN levels were detectable as early as 30’ in plasma and 1h in the urine of patients (162). Attesting the tissue systemic absorption of SFN and ITCs in general, they have been detected in both plasma and synovial fluid of osteoarthritis patients undergoing consumption of GLS-rich diets for 2 weeks (167). On the other hand, the direct delivery of SFN from foods is possible and was demonstrated in recent clinical studies (168, 169) where SFN was shown to be readily bioavailable (170); however, SFN is unstable, requires storage at freezing temperature, and SFN-enriched extracts are difficult to prepare and very expensive (163).

Although most of the research on the biological effects of SFN is focused on cancer because of its effect on cell cycle and apoptosis (171–173), it also regulates bone cells: *in vitro*, SFN inhibits monocyte cell proliferation and osteoclast differentiation in multiple ways, detailed above (100–104), while increases mineralization in mouse MSCs and in an *ex vivo* culture of calvariae explants (97). Notably, in one *in vivo* study the administration of SFN for 5 weeks to normal and ovariectomized mice lead to an approximate 20% increase in bone mass (97), shifting the balance of bone homeostasis and favoring bone acquisition and/or mitigation of bone resorption.

Of note, our group recently demonstrated that GRA obtained from Tuscan black kale promotes osteogenesis in human MSCs, independent of SFN, and this effect is associated to the release of H_2_S and an increased H_2_S uptake inside the cells (98). This is relatively unexpected as GLS have been considered for many years a relatively inert precursor of reactive derivatives ITCs. Although the chemistry underlying this phenomenon is still unclear and will require further investigation, this finding suggests that GLS may exert inherent biological activity based on their capacity to release H_2_S.

**Figure 4:**
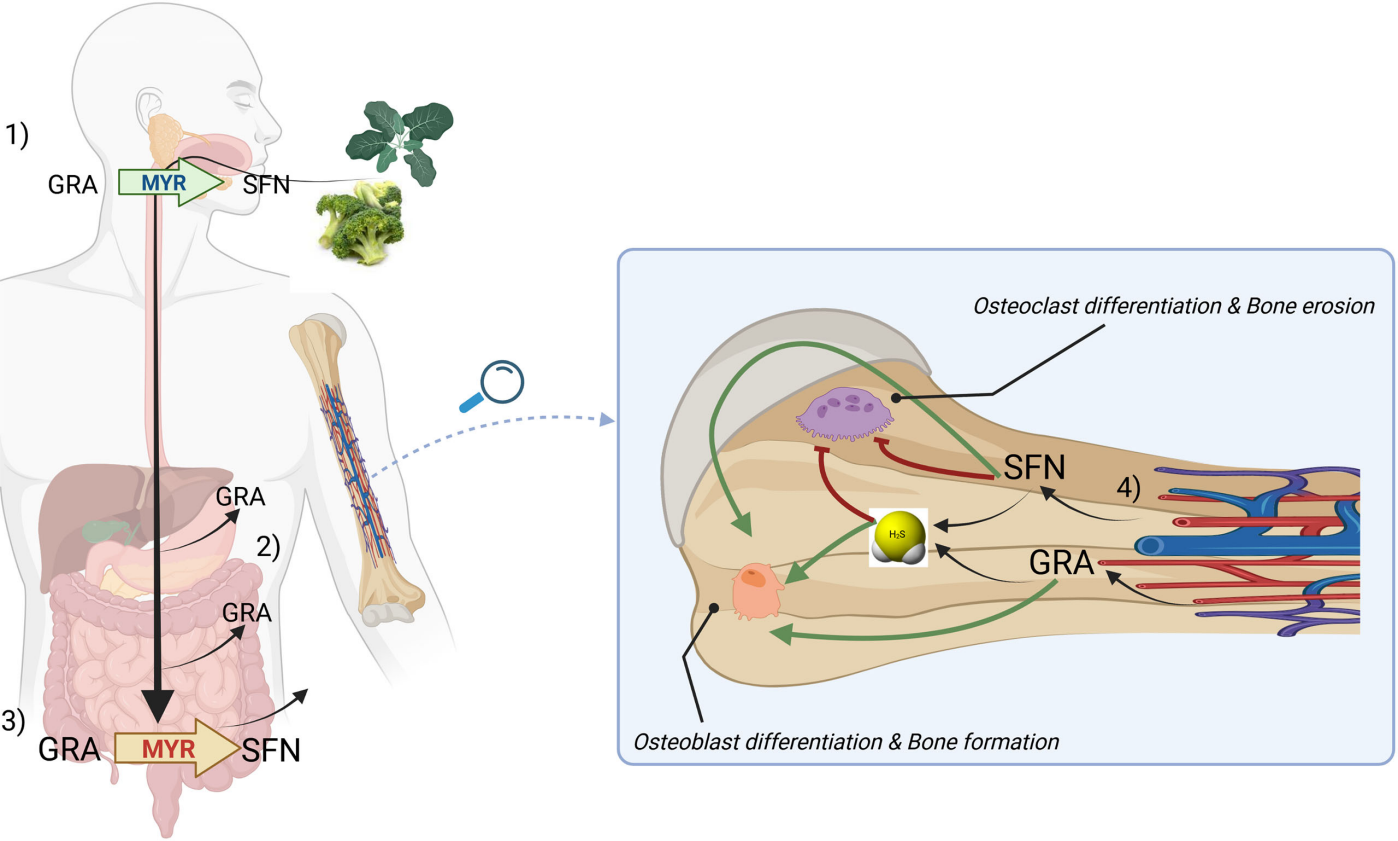
A general model describing the routes of absorption of GRA and SFN and a proposed mechanism of action on bone cells based on H_2_S-release. Briefly, upon chewing of plants belonging to *Brassica* genus, myrosinase (MYR, green) is released and can convert glucoraphanin (GRA) to sulforaphane (SFN) (1). GRA can be adsorbed in the stomach or in the small intestine (2). Microbacterial thioglucosidases (MYR, red) converts GRA to SFN which is further adsorbed in large quantities (3). SFN and GRA are released by circulation in bone tissue where can release H_2_S and exert anabolic and anticatabolic properties on bone cells (4). The mechanism by which H_2_S can be directly released from GRA has not been clarified yet.

As the hydrolytic product of GRA, SFN, had been already shown to inhibit the activity of osteoclast in bone, it can be suggested that the ‘GRA-SFN system’ exerts a beneficial effect on bone both at level of GLS and of its cognate ITC. The routes of absorption of GRA and SFN as well as the proposed mode of action on bone cells is summarized in **Figure 4**.

## Clinical studies

### OSCs and chronic diseases

Despite this review focuses primarily on the skeletal effects of OSCs, much of the clinical research on the health benefits of OSCs is aimed at metabolic or cardiovascular disease and cancer.

Vegetables or extracts rich in OSCs improved dyslipidemia, insulin resistance, hypertension and cardiovascular risk linked to atherosclerotic plaques in human studies.

Among interventional, randomized clinical trials, Jeon et al. evidenced that ethanol extracts from *Brassica rapa*, administrated as a part of the diet of overweight human for 10 weeks, induce a significant increase in the HLDL-cholesterol concentration and a significant reduction in the total cholesterol/HDL-cholesterol ratio, free fatty acid, and adipsin levels (174). A randomized double-blind trial, performed by Bahadoran et. al., investigated the effects of broccoli sprouts powder containing high concentration of SFN for four weeks in type 2 diabetic patients and showed that broccoli sprouts improve insulin resistance by decreasing serum insulin concentration and ‘homeostatic model assessment for insulin resistance’ (HOMA-IR) score (175).

In a prospective cohort study on Australian women aged 70 years and older, without clinical atherosclerotic vascular disease (ASVD) or diabetes mellitus at baseline, Blekkenhorst et al. investigated the occurrence of ASVD‐related deaths during 15 years of follow‐up and correlated it with several dietary intake, through a multivariable‐adjusted model. Among the nutrients tested, intakes of cruciferous and *Allium* vegetables were inversely associated with ASVD mortality supporting the evidence that the effect of increased intake of cruciferous and *Allium* vegetables lowered cardiovascular disease risk (176).

In cancer, treatment with OSCs-rich food showed promising results as chemopreventive.

A placebo double-blind randomized controlled trial on men scheduled for prostate biopsy and treated with broccoli sprout extract (BSE) supplementation (providing SFN and myrosinase) for 4.4 wk, performed by Zhang et. al., showed that BSE supplementation correlated with changes in gene expression but not with other prostate cancer immunohistochemistry biomarkers (173). In a double-blind placebo randomized clinical trial in patients with colorectal adenomas-precancerous lesions of the large bowel treated with aged garlic extract (AGE), Tanaka et al. demonstrated that AGE significantly reduced the size and number of colon adenomas in patients after 12 month (25). Several epidemiological studies showed that SFN consumption has been reported to be associated with a lower risk of cancer development (breast, lung, stomach, esophagus, mammary glands, gastric, colorectal, prostate, skin, head and neck, and liver) (172). In a large cohort study Millen et al. correlated the presence of adenoma with food intake of several fruit and vegetables, as assessed by a food-frequency questionnaire, and showed that onions and garlic were significantly related to lower risk of adenoma (177). Notably, a randomized double-blinded intervention study, performed by Traka et. al., showed that consuming GRA-rich broccoli for 12 months reduced the risk of prostate cancer progression (178). In particular, patients administrated with a weekly portion of soup made from a standard broccoli or 2 experimental broccoli genotypes with enhanced concentrations of GRA, showed dose-dependent attenuated activation of gene expression associated to oncogenic pathways in transperineal biopsies; and an inverse association between consumption of cruciferous vegetables and cancer progression was observed (178).

Overall, these studies highlighted the significant role of diet administration of OSCs in several chronic diseases and substantiate the relevance of creating specific dietary regimen for their prevention.

### OSCs in the prevention of bone loss and skeletal frailty

A few clinical trials or population-based studies have revealed positive relationships between the consumption of vegetables, bone density, muscle strength and fractures in women/men, as summarized in **Table 6**.

**Table 6:** Clinical studies on musculoskeletal effects of OSCs-rich food and extracts.

Molecule tested	Patients data	Mode of administration, concentration, treatments	Main effect	Specific features	Authors	Ref
**Onion**	Perimenopausal and postmenopausal non-Hispanic white women, 50 years and older	Onion consumption ≥ once a day; 3-5 a week; 2 a month to 2 a week, 1 a month or less	Prevention of bone loss	↑ BMD by increased consumption	Matheson et al.	(179)
**Onion juice**	Healthy subjects, male and female, 40-80 years	100 mL of onion juice or placebo for 8 weeks	Decreased bone anabolic markers	↓ALP serum level (commercial kit)	Law et al.	(85)
**Onion juice**	Postmenopausal women	100 mL of onion juice or placebo for 8 weeks	Mild changes in BMD	↓ALP serum levels (commercial kit) Mildly improved BMD (DEXA of the lumbar, right and left hip)	Law et al.	(85)
***Allium* vegetables (onion, leek, and garlic)**	Women, ≥70 years	Habitual intakes of *Allium* intake	Inversely associated with all fractures	Inversely associated with all fractures	Blekkenhorst et al.	(18)
**Cruciferous** **(cabbage, brussel** **sprouts, cauliflower, and broccoli)**	Women aged >70 years	Cruciferous vegetables intake	Inversely associated with all fractures	Inversely associated with all fractures	Blekkenhorst et al.	(18)
**Raw garlic consumption**	28958 patients (males and females)	Habitual intakes of raw garlic	Positive correlation with handgrip strength		Gu et al.	(139)

Analysis of bone mineral density (BMD) by Dual-energy X-ray absorptiometry (DEXA). Measurement of alkaline phosphatase (ALP). ↑ means up-regulation; ↓ means down-regulation.

Matheson et al. used a food frequency questionnaire added to the Nutritional Health and Nutrition Examination Survey (2003–2004) to examine the correlation between habitual consumption of onion over the past 12 months to BMD (N unweighted =507; N weighed =35.7 million). They found that higher consumption of onion increased the BMD by 5% (179). Law et al. administrated onion juice to healthy men and women and post-menopausal women for 8 weeks and investigated the association with bone BMD; the results found that the BMD of 3 postmenopausal women was mildly improved at the end of the treatment (85).

In an intriguing study, Blekkenhorst et al. used a food frequency questionnaire to examine the associations of vegetable and fruit intakes, separately, and specific types of vegetables and fruits with fracture-related hospitalizations in a prospective cohort of elderly women (mean age ≥ 70; n=1468); the authors found that the consumption of vegetable, but not fruit, is associated to a lower incidence of fracture; of note, the habitual consumption of cruciferous vegetables and *Allium* vegetables was significantly inversely associated with all fractures (18); importantly, these results were adjusted for energy intake and physical activity.

In musculoskeletal ageing, sarcopenia and declining physical activity are often associated with osteoporosis as the clinical hallmarks of frailty (180).

Interestingly, a prospective cohort study performed on elderly women (mean age ≥ 70; n=1429) investigated the correlation between vegetable consumption and incident falls-related hospitalization over a time-period of 14 years. The authors found that hospitalizations were lower in participants consuming more vegetables, but the consumption of cruciferous vegetables was most strongly associated with lower falls-related hospitalization (181) and was associated with increased muscle strength.

Finally, cross-sectional study, by Gu et. al., demonstrated a positive correlation between raw garlic consumption, assessed using a food frequency questionnaire, and handgrip strength in both males and females (182). The results were adjusted for age, body mass index, smoking status, alcohol-consumption status, education levels, employment status, household income, family history of diseases (cardiovascular disease, hypertension, hyperlipidemia, and diabetes), metabolic syndromes, physical activity, total energy intake, dietary pattern, onion intake. Although this study did not directly assess indexes of bone quantity, it supports an overall protective effect of OSCs-rich vegetables on the musculoskeletal system (181).

## Perspectives and challenges

The present literature revision stems from the increasing appreciation of the link between dietary habits, and particularly the use of phytochemicals, and bone health. We show that a growing body of evidence supports a beneficial effect of dietary OSCs on skeletal health. Of note, although a few population-based studies offer interesting clues on the clinical relevance of OSCs-rich vegetables for the prevention of bone fragility (18, 85, 179, 183), no clinical studies have been performed yet to specifically address the potential protective role of OSCs against osteoporosis or bone fractures; this goal would require a study design including a controlled intake of OSCs-rich nutrients for long time-periods and/or the evaluation of purified OSCs molecules.

The ability of OSCs to work a as dietary source of the bioactive molecule H_2_S provide interesting future perspectives. OSCs-rich vegetables appear as the ideal candidate for clinical investigations on whether nutrients rich in sulfur can affect the pool of circulating reactive sulfur species (RSS), which include H_2_S; this may have a broad implication for the prevention of those pathologies, sometimes referred to as ‘H_2_S-poor diseases’, where the onset of the disease was associated to a lower systemic concentration of RSS compared to healthy controls. Increasing systemic RSS levels may also have important implication for bone-wasting diseases such as osteoporosis: indeed, animal studies have established that the bone loss associated to estrogen deficiency or to corticosteroid therapy is associated to a low systemic level of H_2_S (157, 159). However, these preclinical data still await confirmation in observational clinical studies in humans. To obtain reliable data on this topic, it will be critical to include in the study design a robust analytical methodology to quantitatively measure the different sulfur species in human serum or plasma since they may hold different importance in different pathologies (184, 185) and the high reactivity of these gaseous molecules implies a complex chemistry (186).

Further investigations may be addressed to the evaluation of the effect of these compounds on the gut-bone axis. OSCs show a considerable ability to modulate the gut microbiome and its secondary metabolites (187–190) and to mitigate the gut-based inflammatory response; given the paramount importance of metabolites and cytokines originated from the gut on the regulation on bone metabolism (191), it is conceivable that dietary OSCs may modulate the bone-bioactive components of the microbiota.

In the end, it is apparent that members of the OSCs family of phytochemicals affect bone homeostasis in several ways and may provide new insights into the potential bone health benefits of plant-derived food and leading to a more effective prevention of osteoporosis by non-pharmacological tools. This review may be useful to fuel clinical trials that may use a robust set of outcome measurements, aiming at assessing both bone quantity and bone quality before and after specific nutrition protocols; correlation between nutrients intake, H_2_S blood levels and bone status would help to define preventive/clinical dietary protocols for patients with an increased risk of bone fragility.

## Author contributions

FG and LG contributed to the conception and design of the review. FG ad LG wrote the first draft of the manuscript. All authors contributed to manuscript revision, read, and approved the submitted version.

## Conflict of interest

The authors declare that the research was conducted in the absence of any commercial or financial relationships that could be construed as a potential conflict of interest.

## Publisher’s note

All claims expressed in this article are solely those of the authors and do not necessarily represent those of their affiliated organizations, or those of the publisher, the editors and the reviewers. Any product that may be evaluated in this article, or claim that may be made by its manufacturer, is not guaranteed or endorsed by the publisher.

## Glossary

**Table:**

ACP5	Acid phosphatase 5, tartrate resistant
AG	Age-associated
AGE	Aged garlic extract
AITC	Allyl isothiocyanate
ALP	Alkaline phosphatase
ASCOs	*S*-alk(en)yl cysteine sulfoxides
AS	Allyl sulfide
ASVD	Atherosclerotic vascular disease
Atg5	Autophagy related 5
Atg12	Autophagy related 12
BITC	Benzyl isothiocyanate
BM	Bone marrow
BMD	Bone mineral density
BMM	Bone marrow macrophages
BMMSCs	Bone marrow-derived mesenchymal stem cells
BMP	Bone morphogenetic protein
BS/BV	Bone surface/bone volume
BSE	Broccoli sprout extract
BSP	Bone sialoprotein
BV	Bone volume
BVF	Bone volume fraction
BV/TV	Bone volume / trabecular volume
CAT	Catalase
CBS	Cystathionine beta synthase
Clcr	Calcitonin receptor-like receptor
CCK-8	Cell counting kit-8
c-fms	Colony-stimulating factor-1 receptor
Col I	Collagen I
CSE	Cystathionine-γ-lyase
CTR	Calcitonin receptor
CTX-I	Serum type I collagen breakdown product
CTSK	Cathepsin K
DADS	Diallyl disulfide
DATS	Diallyl trisulfide
DC-STAMP	Dendritic cell-specific transmembrane protein
DEXA	Dual-energy X-ray absorptiometry
Dlx5	Distal-Less Homeobox 5
DPDS	Dipropyl disulfide
ECM	Extracellular matrix
ER	Erucin
ERK	Extracellular signal-regulated kinase
EZ4U	3-(4,5-Dimethylthiazol-2-yl)-2,5-diphenyltetrazolium bromide-like assay
FcRγ	Fc receptor standard g chain
FOXO	Forkhead box O
GCLC	Glutamate cysteine ligase catalytic subunit
GCLM	Glutamate-cysteine ligase modifier subunit
GH	Growth hormone
GLS	S-β-thioglucoside N-hydroxhysulfates; glucosinolates
GPCS	γ-glutamyl-*trans*-*S*-1-propenyl-L-cysteine sulfoxide
GRA	Glucoraphanin
GSAC	γ-glutamyl-*S*-allyl-L-cysteine
GSH	Glutathione
HBITC	4-hydroxybenzyl isothiocyanate
HO1	Heme oxygenase-1
HOMA-IR	Homeostatic Model Assessment for Insulin Resistance
H_2_S	Hydrogen sulfide
IGF-1	Insulin-like growth factor 1
ITCs	Isothiocyanates
JAK2	Janus Kinase 2
JNK	Jun N-terminal kinases
KEAP-1	Kelch-like erythroid-cell-derived protein with CNC homology (ECH)-associated protein
LC3-II	Microtubule-associated protein 1A/1B-*light* chain 3
LPS	Lipopolysaccharide
MAPK	Mitogen-activated protein kinase
M-CSF	Macrophage colony-stimulating factor
MMP-9	Matrix metallopeptidase 9
MDA	Malondialdehyde
MSC	Mesenchymal stromal cells
MTT	3-(4,5-Dimethylthiazol-2-yl)-2,5-diphenyltetrazolium bromide
NFATc1	Nuclear factor-activated T cells c1
NF-κB	Nuclear factor kappa-light-chain-enhancer of activated B cells
NQO1	NAD(P)H: quinone oxidoreductase 1
NOX-1	NADPH oxidase 1
NRF2	Nuclear factor erythroid-derived 2-related factor 2
OCN	Osteocalcin
OC-STAMP	Osteoclast-stimulatory transmembrane protein
OP	Osteoporosis
OPG	Osteoprotegerin
OPN	Osteopontin
OSCAR	Osteoclasts-specific activating receptor
OSCs	Organosulfur compounds
OSX	Osterix
P1NP	Procollagen 1 intact N-terminal propeptide
PeCSO	γ-glutamyl-propenyl-L-cysteine sulfoxide
pNPP	*p*-nitrophenyl phosphate
PPAR-γ	Proliferator-activated receptor-γ
PRDX-1	Peroxiredoxin 1
PTH	Parathormone
RAC1	RAC family small GTPase 1
RANK	Receptor activator of nuclear factor κ B
RANKL	Receptor activator for nuclear factor κ B ligand
RUNX-2	Runt-related transcription factor 2
ROS	Reactive oxygen species
RSS	Reactive sulfur species
SAC	*S*-allylcysteine
SAMC	*S*-allylmercaptocysteine
SAMG	*S*-allylmercaptoglutathione
SFN	Sulforaphane
SIRT	Sirtuin
SMAD-1	SMAD family member 1
STAT3	Signal transducer and activator of transcription 3
Tb.N	Trabecular number
Tb.Th	Trabecular thickness
Tb.Sp	Trabecular space
TRAF-6	Tumor necrosis factor receptor-associated factor 6
TRAP	Tartrate-resistant acid phosphatase
TRAP5b	Tartrate-resistant acid phosphatase 5b
VOSCs	Volatile organosulfur compounds
WISP-1	WNT1-inducible-signaling pathway protein 1
Wnt	Wingless/Integrated
WST-8	Water-soluble tetrazolium-8
